# Single-Particle View of Doping-Controlled Charge Recombination in BiVO_4_

**DOI:** 10.1021/acs.nanolett.6c03155

**Published:** 2026-10-01

**Authors:** Sean McCoy, Devon Stritesky, Liam Todd, Marissa Ashworth, Mahdi Hesari

**Affiliations:** † Department of Chemistry, 14828 State University of New York (SUNY) Oswego, Oswego, New York 13126, United States; ‡ Department of Computer Science and Engineering, 14828 State University of New York (SUNY) Oswego, Oswego, New York 13126, United States; § Department of Atmospheric and Geological Sciences, 14828 State University of New York (SUNY) Oswego, Oswego, New York 13126, United States

**Keywords:** Single-particle photoluminescence, Confocal microscopy, BiVO_4_ particle, Chemical doping

## Abstract

Chemical doping of semiconductors has been widely used to alter and enhance the photochemistry of materials. More specifically, the nature of the doping element(s) influences the inherent electronic and structural properties of semiconductors. The morphology of the semiconductor particles can further impact the photocatalytic performance of the materials. We report here that solo- and codoping of truncated pyramidal BiVO_4_ with phosphorus and nitrogen affects the photoluminescence (PL) pattern and enhances the emission intensity, as observed using confocal fluorescence microscopy. The PL emission has been shown to increase with doping in P:BiVO_4_, N:BiVO_4_, and P:N:BiVO_4_ by 6.8%, 10.8%, and 19.2%, respectively, relative to that of BiVO_4_. Our subparticle analyses revealed a trend of corner edge > top edge > side edge. We demonstrated that codoping diminishes the sensitivity of PL to the particle volume, reducing the impact of structural heterogeneity in BiVO_4_.

## Graphical abstract

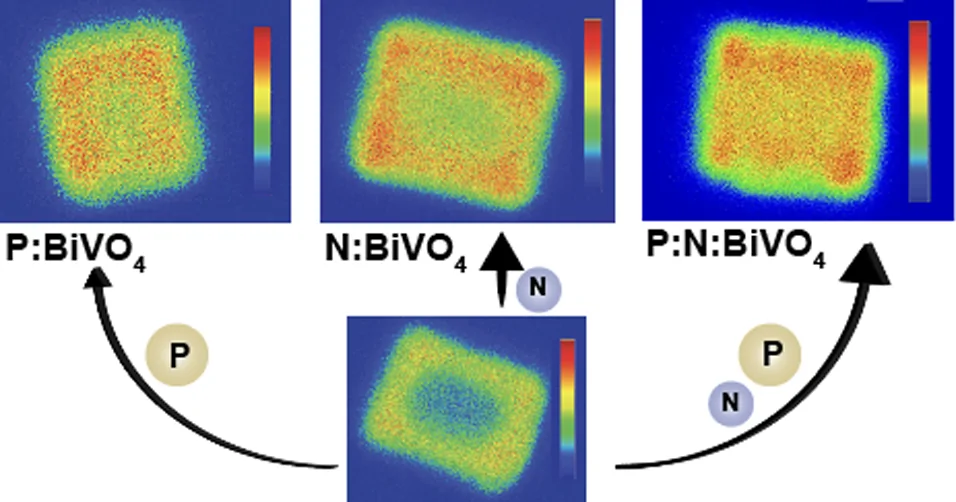

Semiconductor materials have received significant attention across a range of applications, including microchips, sensors, and green energy technologies such as hydrogen production. In particular, semiconductors play a crucial role as photocatalysts and photoelectrocatalysts for light-to-chemical conversion processes.^1^ Thus, the photochemistry of semiconductors provides essential information for their applications in optical displays,^2^ photocatalysis,^3,4^ photoelectrocatalysis in solar devices,^5,6^ and sensing.^7^ Among various semiconductor materials, bismuth vanadate (BiVO_4_) has demonstrated a strong performance and gained attention for its photophysical properties, including a relatively short band gap of ∼2.54 eV.^8–10^ BiVO_4_ has been achieved in film^11–13^ or particulate^14–17^ forms and shows promising performance in energy conversion processes, including the splitting of water into hydrogen and oxygen.^13,18,19^ Various strategies have been developed to improve the performance of BiVO_4_. A guest element, i.e., dopant, enhances the overall properties of the semiconductor material by increasing charge separation or improving conductivity. Metallic dopants such as calcium,^20^ iron,^21^ nickel,^22^ tungsten,^23^ molybdenum,^24^ and gadolinium^25^ have been incorporated into the BiVO_4_ structure for hydrogen peroxide production, water decontamination, and water splitting. In these examples, the doping agents were considered to alter the valence band (VB) or conduction band (CB) to improve the energy alignment and produce the desired products, i.e., O_2_, H_2_, or H_2_O_2_. Using nonmetallic elements, e.g., phosphorus and nitrogen, as dopants also influences the photophysics of BiVO_4_.^26–29^

The presence of phosphorus in the crystal structure influences the material’s electronic structure, broadening the VB and therefore shortening the band gap of P:BiVO_4_ relative to that of BiVO_4_.^29^ It has been shown that an increase in the oxygen vacancy (O_v_) density creates additional intraband states, thereby facilitating photoluminescence (PL) emission.^30^ Wang and colleagues^31^ demonstrated that phosphorus induces band-structure reconstruction and increases O_v_ formation. Moreover, phosphorus–oxygen–metal could also influence the structural distortion.

Choi and colleagues^27^ demonstrated that nitrogen incorporation in BiVO_4_ reduces the band gap and enhances light absorption while increasing O_v_ and charge mobility. The Chen group^32^ also reported that nitrogen doping increases the {010} donor density and improves photoelectrochemical water splitting in N:BiVO_4_ relative to BiVO_4_. Recently, Wiktor and colleagues, using hybrid density functional theory (DFT), showed that surface nitrogen in N:BiVO_4_, along with O_v_ introduced by doping, synergistically improves the oxygen evolution reaction performance of BiVO_4_.^33^ Phosphorus doping has shown comparable benefits: Fu and colleagues^34^ found that P enhances the O_v_ concentration and charge mobility and slightly narrows the band gap.

Both nitrogen and phosphorus act as soft dopants, reinforcing the intrinsic n-type character of BiVO_4_. However, systematic studies of how these dopants influence their photophysical properties remain limited. More specifically, there have been no reports of incorporating both N and P into the structure of BiVO_4_. Moreover, the influence of doping on the photochemistry of particulate BiVO_4_ crystals remains poorly understood.

Many studies have provided insights into the structure and function of BiVO_4_ films^13^ and particulate forms.^32^ Confocal fluorescence microscopy (CFM) was used to investigate the PL pattern for BiVO_4_ under photoelectrochemical conditions.^35^ Mao et al.^36^ conducted single-particle PL measurements of flake-BiVO_4_ and truncated bipyramid-BiVO_4_, suggesting that t-BiVO_4_ has a higher charge recombination rate than f-BiVO_4_. Zhang and colleagues^37^ employed CFM to investigate O_v_ on single BiVO_4_ particles before and after hydrogen treatment, concluding that PL emission originates from O_v_ in BiVO_4_ and establishing a positive correlation between defects and bound-exciton emission. They also successfully employed single-particle PL spectroscopy to explore photoinduced charge transfer in TiO_2_ decorated with BaTiO_3_ ^38^ and monitor charge transfer in SrTiO_2_/TiO_2_ epitaxial heterojunctions.^39^ They observed that the PL intensity of SrTiO_2_/TiO_2_ exceeds that of TiO_2_ at the edges and corners, indicating enhanced carrier mobility at the TiO_2_{001}/SrTiO_3_ interfacial heterojunction. The Majima group conducted single-particle PL measurements of an anatase TiO_2_ particle using CFM to investigate the effect of O_v_ for photocatalytic hydrogen generation.^40^ Their results revealed that the surface junction, i.e., edge or corner, of the particle has a longer emission lifetime than the center.^41^ Yang and colleagues utilized CFM to investigate various perovskite materials.^42,43^ They quantified the anion-exchange kinetics of single-particle halide perovskites.^42^

We prepared a series of BiVO_4_ truncated bipyramidal particles doped with nitrogen (N:BiVO_4_), phosphorus (P:BiVO_4_), and codoped phosphorus–nitrogen (P:N:BiVO_4_) and investigated their solid-state PL using CFM at the single-particle level relative to undoped BiVO_4_. We aimed to investigate how N and P, as well as P:N-doping BiVO_4_, influence the photophysics of truncated bipyramidal particles. The results revealed that the doping elements alter the PL in intensity and pattern. Furthermore, our single-particle measurements revealed that the particle volume dependence can be tuned and overcome through a doping strategy.

The synthesis procedures for the BiVO_4_ materials and confocal microscopy details are provided in the Supporting Information. Scanning electron microscopy (SEM) images of the ensemble samples (Figure S1) confirm the truncated pyramid morphology of BiVO_4_, P:BiVO_4_, N:BiVO_4_, and P:N:BiVO_4_. Within the truncated pyramid shape of BiVO_4_, the crystal facets, i.e., {010} basal and {110} lateral, are energetically favorable sites for the localization of photogenerated electrons and holes.^16^ More importantly, the boundaries between these facets serve as active sites for photocatalysis and photoelectrocatalysis.^32,44^ The elemental compositions of the materials, i.e., undoped and doped, were confirmed using SEM–energy-dispersive X-ray spectroscopy (SEM–EDX). The greater penetration depth of SEM–EDX (e.g., 15 μm) has enabled elemental composition extraction for BiVO_4_, P:BiVO_4_, N:BiVO_4_, and P:N:BiVO_4_. The results indicated an average P doping of 0.3 ± 0.03% and a N doping of 4 ± 0.2% in P:BiVO_4_, N:BiVO_4_, and P:N:BiVO_4_ (Table S1).

Figure S3 shows high-resolution X-ray photoelectron spectroscopy (XPS) of BiVO_4_, P:BiVO_4_, N:BiVO_4_, and P:N:BiVO_4_, confirming the presence of phosphorus and nitrogen in the doped BiVO_4_. The high-resolution XPS analysis of P:BiVO_4_ (Figure S3G) shows a P 2p peak at 132.6 eV, attributed to P^5+^ in the PO_4_^3–^ unit within the crystal structure.^29^ The XPS results further confirm that the introduction of phosphoric acid altered the vanadium (V^5+^) mole percentage and led to the formation of V^4+^. The nitrogen-doped BiVO_4_ XPS spectrum at 399.9 eV also supports the presence of N 1s within the crystals (Figure S3K).^45,46^ The XPS spectra of P:N:BiVO_4_, however, show only a peak associated with nitrogen. This could be due to (i) higher surface nitrogen doping in P:N:BiVO_4_ and (ii) the shorter X-ray penetration depth of XPS (∼5 nm) relative to the electron beam penetration of EDX (3–5 μm). In addition, the O 1s spectra in all samples, at about 531–532 eV, correspond to oxygen lattice (O_L_, ∼529 eV), O_v_ (∼530 eV), and oxygen adsorbed (O_ads_, ∼531 eV).^47^ These results support the formation of O_v_, which is common upon doping of BiVO_4_.^22,46,48^

Figure 1A shows the Tauc plots of BiVO_4_, P:BiVO_4_, N:BiVO_4_, and P:N:BiVO_4_ materials. There are slight differences in the optical characteristics between undoped and doped BiVO_4_ materials. This represents the energy differences between the CB and VB of the various materials. The solid-state ensemble PL spectra (Figure 1B) of BiVO_4_, P:BiVO_4_, N:BiVO_4_, and P:N:BiVO_4_ in the range of 488.057–700.15 nm (2.539–1.7702 eV) were obtained using a 488 nm laser and revealed a similar emission energy at 510 and 532 nm and a broad emission band starting around 545.8–700 nm (2.27–1.77 eV). The peak at 2.43 eV is attributed to V–O symmetric Raman modes (and O_v_).^49^ The other peaks are related to the sub-band gap and/or defect-state (trap-state) emissions and elemental-doping emissions (Figure 1F). The PL spectroscopy did not show noticeable spectral shifts, likely due to particle heterogeneity that averages out individual responses, which potentially could be resolved only at the single-particle level. Furthermore, consistent with previous reports,^27,29^ N and P substitute O and V sites, respectively, and contribute primarily to nonradiative recombination pathways rather than radiative emission, leaving the PL peak position unchanged.

**Figure 1:**
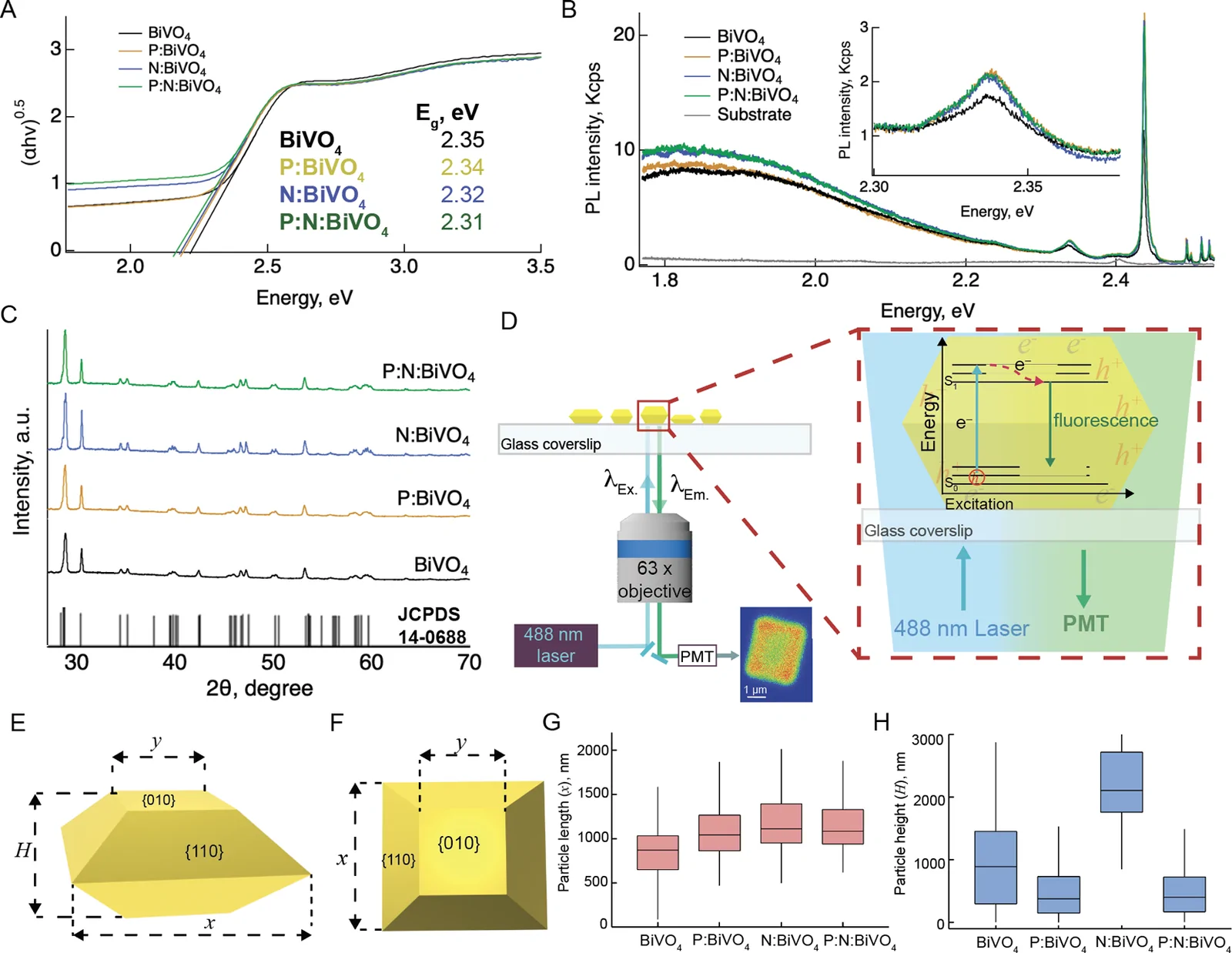
Characterization of BiVO_4_ crystals and single-particle confocal fluorescence imaging. (A) Tauc plot of the solid-state film. (B) PL spectroscopy. (C) Powder X-ray diffraction patterns of BiVO_4_ crystals. (D) Schematic of confocal fluorescence and PL of particulate BiVO_4_. (E and F) 3D image or schematic of the crystals demonstrating the {010} basal and {110} lateral lengths. (G) Length and (H) calculated height of the BiVO_4_ particle. The peak at 2.43 eV in panel B is attributed to V–O symmetric Raman modes (and O_v_).^49^

The X-ray diffraction patterns of the undoped and doped BiVO_4_ samples are similar, supporting fine crystal morphology (Figure 1C). Figure 1D shows a single-particle confocal setup used to collect single-particle PL from BiVO_4_ materials (more details are provided in the Supporting Information). Parts E and F of Figure 1 display a schematic of truncated bipyramids of BiVO_4_ in which we measured the *x* and *y* dimensions of each particle and calculated the height (*H*) according to the literature (see the Supporting Information).

Figure 2 displays bright-field microscopy, SEM, and confocal PL images of BiVO_4_, P:BiVO_4_, N:BiVO_4_, and P:N:BiVO_4_ single particles. The bright-field images (Figure 2A–D) reveal an opaque dark and light-yellow contrast, representing {110} and {010}, i.e., lateral and basal facets. To further correlate and understand the fine features of confocal images of the same particles, SEM images were collected and are shown in panels E–H for BiVO_4_, P:BiVO_4_, N:BiVO_4_, and P:N:BiVO_4_, respectively. The solid and dashed lines delineate the boundary between the {110} and {010} facets and correspond to bright-field optical images.

**Figure 2:**
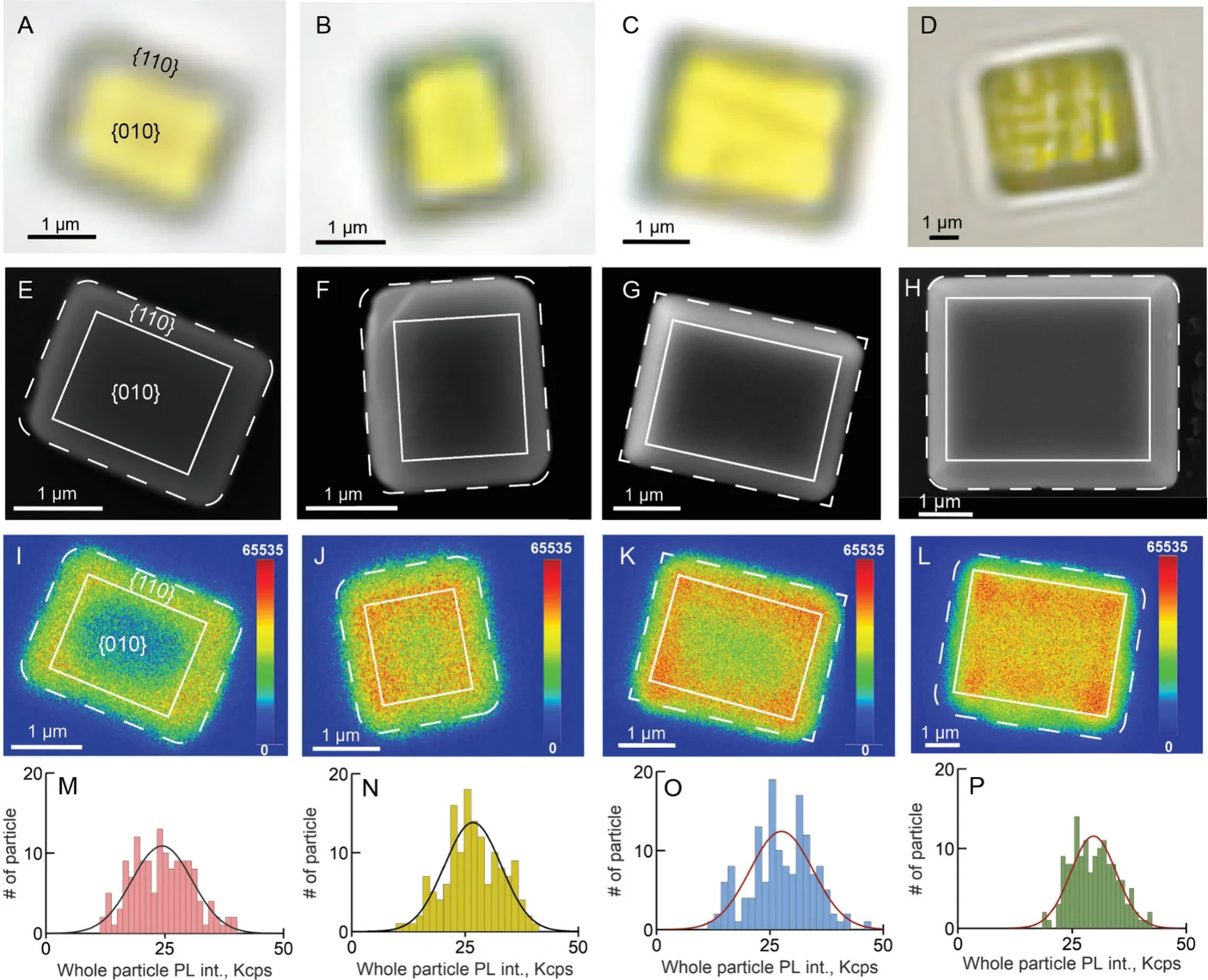
Single-particle microscopy of BiVO_4_ crystals. (A–D) Bright-field microscopy, (E–H) SEM, and (I–L) confocal PL spectroscopy of undoped BiVO_4_, P:BiVO_4_, N:BiVO_4_, and P:N:BiVO_4_ single crystals. Gaussian curve-fitted histograms (bin size: 25) of the whole-particle PL intensity of 144 single particles of (M) BiVO_4_, (N) P:BiVO_4_, (O) N:BiVO_4_, and (P) P:N:BiVO_4_.

Figure 2I shows the confocal images of the same single BiVO_4_, shown in panels A and E, in which the {010} facet reveals lower PL emissions and the {110} facet higher PL emissions. This pattern resembles the PL pattern of single BiVO_4_ in the absence of an electrochemical bias reported by Tachikawa et al.,^35^ where more PL intensity was observed at the lateral facets. Zhang et al. also observed a similar PL pattern for BiVO_4_ at the single-particle level by using confocal microscopy.^50^ This suggests a higher probability of photogenerated electron–hole recombination over the {110} facets, where holes reside. This has been correlated to the preferred location of O_v_, which acts as an electron capture center where they can recombine with holes.^35,37^ Moreover, the facet junctions could act as carrier funnels where photogenerated electrons and holes migrate toward and recombine. This provides a unique opportunity to alter the PL intensity and pattern of BiVO_4_ crystals by changing (1) O_v_,^37^ (2) the crystal shape,^36^ and (3) the electron density. The latter could play a crucial role in enhancing the PL emission and pattern formation in BiVO_4_. To evaluate this opportunity, we studied the PL emission patterns of the P:BiVO_4_, N:BiVO_4_, and P:N:BiVO_4_ single crystals.

Figure 2J displays a confocal image of P:BiVO_4_, in which the image reveals an intensity enhancement and emission pattern dominantly concentrated at the {110} and {010} facets’ junctions. This visual observation represents the effect of phosphorus incorporation on the PL behavior of individual P:BiVO_4_ particles.

To further understand the effect of the size and intrinsic nature of the doping element on the solid-state PL of BiVO_4_, the confocal PL microscopy of N:BiVO_4_ was studied. Figure 2K shows an exemplary single N:BiVO_4_ particle’s confocal PL image. One may notice the enhanced PL intensity over the particle with a pattern similar to that of P:BiVO_4_, where the basal {110} and lateral {010} facets’ edges are more emissive than the basal facet. More interestingly, a substantial PL intensity enhancement and a change in the pattern of PL emission were observed in P:N:BiVO_4_-codoped crystals. Figure 2L represents a single P:N:BiVO_4_ particle in which a major enhanced PL can be seen over the {110} basal facet.

To further elucidate the confocal PL microscopy of BiVO_4_ single crystals and characterize the statistically reliable ensemble behavior of BiVO_4_, confocal images of 144 individual particles of BiVO_4_, P:BiVO_4_, N:BiVO_4_, and P:N:BiVO_4_ were analyzed. The mean PL intensity was obtained by integrating the observed PL of each particle (see the Supporting Information).

Statistical analyses were performed using Minitab. The normality of each data set (*n* = 144), including PL intensities of BiVO_4_ materials, was evaluated using the Kolmogorov–Smirnov test. All data sets yielded *p* values greater than 0.05, indicating that the data followed a normal distribution (Figure 2L–O). The corresponding means and standard deviations were subsequently used to generate Figure 3A. The same normality assessment was applied to the single-particle data sets, and the resulting means and standard deviations are presented in the bar plots of Figure 4.

**Figure 3:**
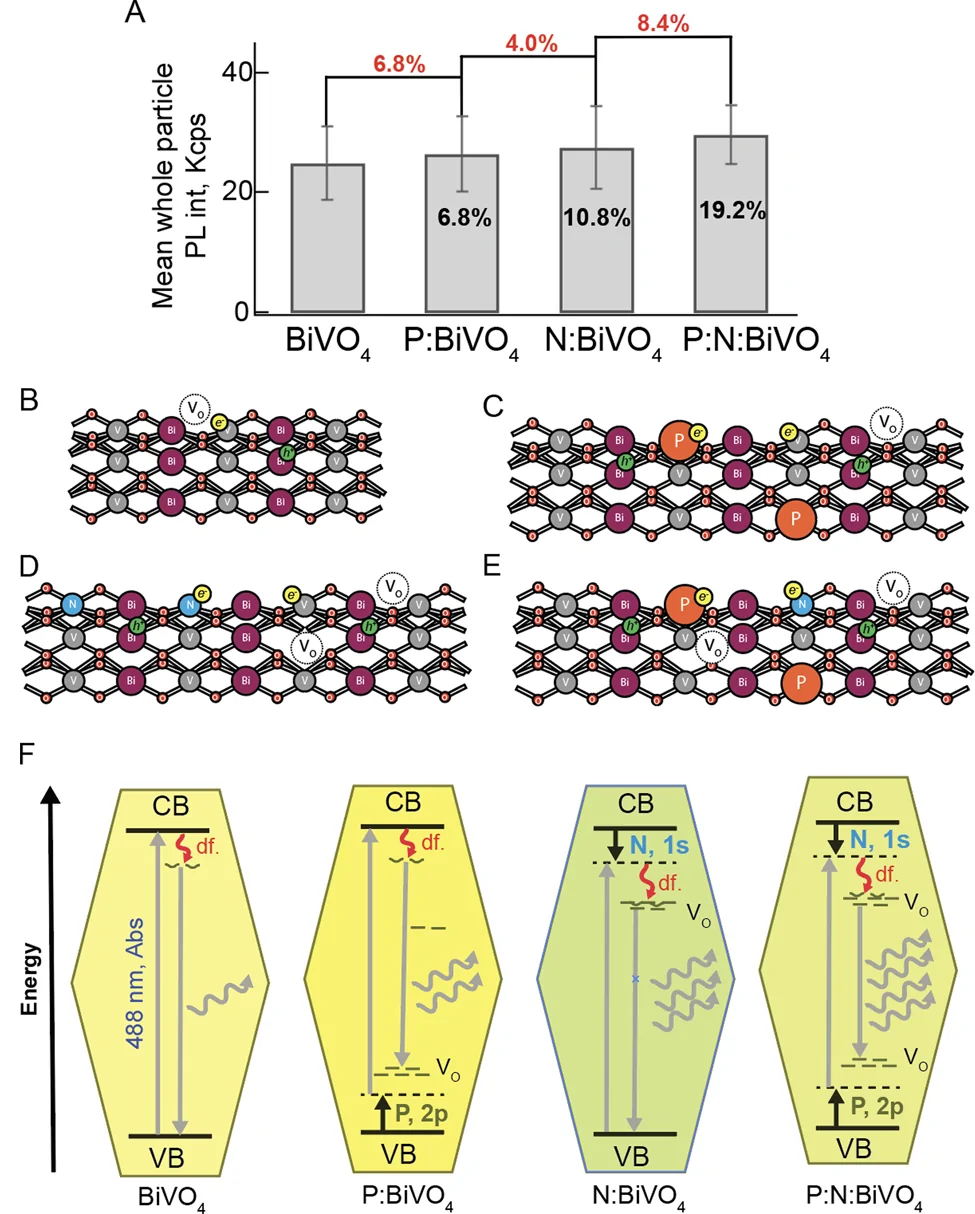
(A) Bar plot summarizing the mean PL intensity extracted from Figure 2M–P. The top bar and inside bar numbers represent the PL intensity changes with respect to each other and with undoped BiVO_4_. The error bars represent the standard deviation of the dispersion. Schematic representation of the atomic structure of the {010} facet surface of (B) BiVO_4_, (C) P:BiVO_4_, (D) N:BiVO_4_, and (E) P:N:BiVO_4_. (F) Schematic energy diagram of undoped and doped BiVO_4_, demonstrating the influence of dopants on the alteration of the energy band gap, as well as the presence and role of the O_v_ and defect states acting as the recombination sites.

**Figure 4:**
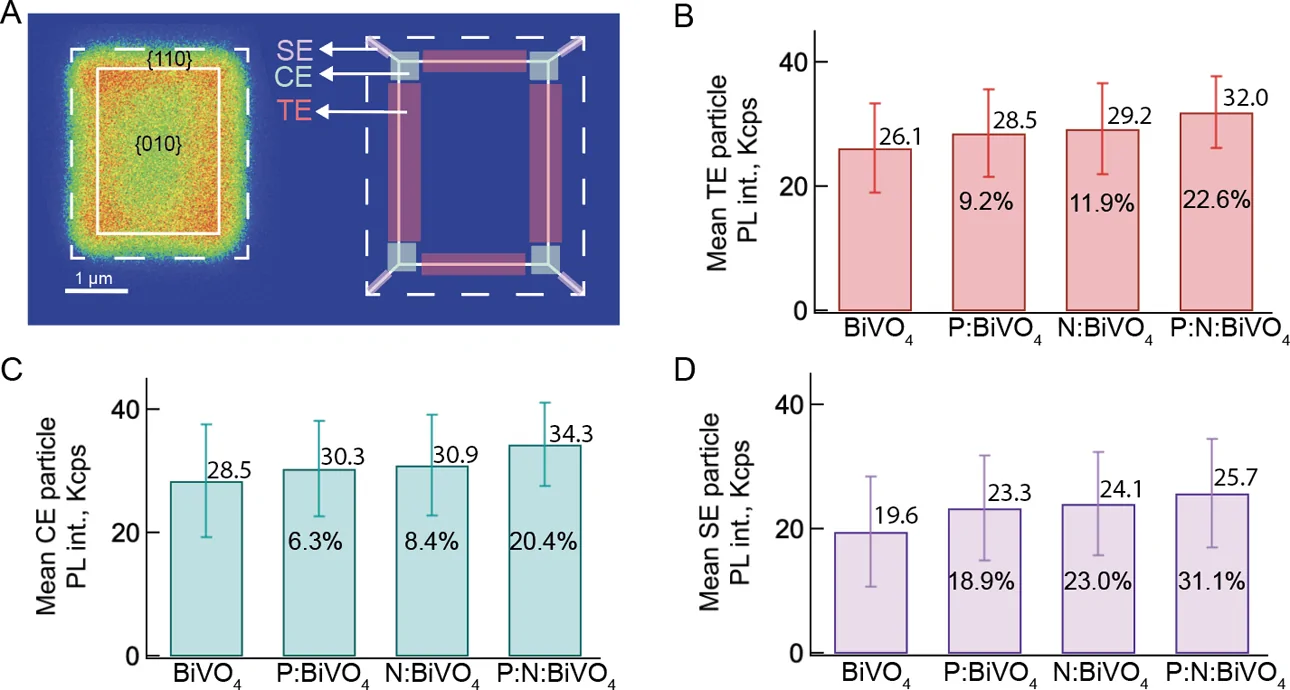
Dissected single-particle confocal fluorescence of BiVO_4_ particles. (A) Exemplary P:N:BiVO_4_ single particle and schematic of the dissected regions of the solid-state PL of the particles. Bar plot of the mean intensity of the dissected area for (B) center edges, (C) top edges, and (D) side edges of 144 single BiVO_4_, P:BiVO_4_, N:BiVO_4_, and P:N:BiVO_4_. The bar colors represent the dissection colors in the right section of panel A. The reported errors represent the average dispersion.

Differences among the PL intensities of BiVO_4_, N:BiVO_4_, P:BiVO_4_, and P:N:BiVO_4_ were evaluated using a one-way ANOVA. The analysis yielded a *p* value of 4.7 × 10^–11^ ≈ 0.00, which is well below the significance threshold of 0.05. This result indicates statistically significant differences among the groups and further supports the conclusions. Figure S8B shows a wide-field confocal image, collected under the same conditions as the individuals, including several particles, representing the heterogeneity among particles. Due to the random dispersion of particles, it is plausible to find adjacent or nearby particles. Figure S9 shows bright-field and confocal images of nearby particles of P:BiVO_4_ at different orientations. There is no obvious and substantial particle-to-particle influence.

Figure 2M represents a histogram of the whole-particle mean PL intensity of BiVO_4_ particles. The histogram was fitted with a Gaussian function (eq 1).

1 $$y=y_{0}+A\;\exp\left[-{\left(\frac{x-x_{0}}{\mathrm{width}}\right)}^{2}\right]$$

where *x*_0_ represents the mean PL intensity of the BiVO_4_ population. The extracted mean whole-particle PL intensity is 24.9 ± 6.1 Kcps. Figure 2N shows the resulting P:BiVO_4_ curve-fitted histogram, where the mean PL intensity is 26.5 ± 6.9 Kcps. This is 1.7 Kcps higher than the BiVO_4_ particles’ PL intensity, which is 6.8% PL enhancement. The observed PL intensity enhancement in P:BiVO_4_ can be attributed to soft doping and the replacement of V^4+^ by P^5+^, which, in turn, leads to the generation of O_v_ within the crystal structure. DFT calculations by Ng and colleagues^29^ proposed that the replacement of intrinsic vanadium­(IV) by phosphorus influences the Fermi level of BiVO_4_. Here, the P to P+V ratio is 1.07%, according to SEM–EDX and XPS analysis results.

The mean PL intensity of 144 single N:BiVO_4_ particles (Figure 2O) is 27.6 ± 6.9, which is 10.8% higher than undoped BiVO_4_ and 4% relative to that of P:BiVO_4_. The observed PL enhancement could be related to both electronic and structural changes due to the presence of nitrogen atoms in N:BiVO_4_, as well as the formation of O_v_, which could act as recombination sites.

The whole-particle mean PL intensity of the same population (*n* = 144) of P:N:BiVO_4_ single particles reveals an average of 29.7 ± 4.9 Kcps (Figure 2P). In fact, the PL intensity of the P:N:BiVO_4_ crystals shows 19.2% and 8.4% enhancements relative to BiVO_4_ and N:BiVO_4_, respectively. The superior enhancement in P:N:BiVO_4_ can be attributed to the synergistic effect of the presence of nitrogen, phosphorus, and a higher density of O_v_ in the particles’ population. Parts B–D of Figure 3 display schematic representations of the atomic structures of BiVO_4_ and P:BiVO_4_, N:BiVO_4_, and P:N:BiVO_4_, where O_v_ and the replacement of O with P and N are demonstrated. The energy band gaps (Figure 3F) upon doping with phosphorus show both a lowering of the energy band gap and O_v_ playing a role in the PL of P:BiVO_4_ relative to BiVO_4_ particles. These schematics represent plausible mechanisms of how doping and the presence and copresence of phosphorus and nitrogen contribute to the energy band gap and light absorption efficiency and, more importantly, enhance the density of O_v_ within the P:N:BiVO_4_ crystals. The resulting PL emission reveals higher PL intensity and the recombination site pattern.

Choi and colleagues demonstrated that mildly annealing the BiVO_4_ film in a N_2_ stream produces nitrogen-doped BiVO_4_, which effectively reduces the band gap, introduces O_v_ sites, and enhances charge-carrier mobility.^27^ They highlighted that increasing the number of O_v_ improves photon absorption. Irani et al.^45^ showed that nitrogen incorporation into the BiVO_4_ structure leads to the formation of a localized state about 0.1 eV below the CB. Figure 3D illustrates the plausible nitrogen replacement within the N:BiVO_4_ structure, and Figure 3F displays energy and O_v_ effects on the PL performance of the material.

In intrinsic BiVO_4_ crystals, surface terminations, including V–O, Bi–O, and V–O–V bonds, display an inhomogeneous distribution dictated by the local composition and structure within individual particles.^51^ The {010} and {110} facets are inherently V-rich and Bi-rich, respectively, driving spatially selective charge-carrier separation: photogenerated electrons preferentially accumulate on {010} facets, while holes migrate to {110} facets.^16,52^

This facet-selective behavior can be systematically modified through doping. Here, phosphorus was introduced via hydrothermal synthesis to partially substitute vanadium, while nitrogen was incorporated through high-temperature annealing, affecting both the V and/or Bi sites. DFT calculations by El Ouardi et al.^53^ support this structural interpretation, showing that N incorporation replaces Bi–O bonds with Bi–N bonds, accompanied by an increase in bond length. Importantly, elemental doping, whether chemical (P) or thermal (N), promotes O_v_ formation, and codoping with both P and N produces a synergistic enhancement of the vacancy density. Because PL emission scales with the dopant concentration and O_v_ population, P:N:BiVO_4_ exhibits significantly stronger PL emission from the {010} facet compared to undoped BiVO_4_, reflecting a pronounced redistribution of surface electronic states driven by codoping. The curve-fitted high-resolution XPS spectra (Figure S3) revealed that there is (i) a fraction of V^4+^ formation that has been shown to assist O_v_ in acting as recombination and radiative centers rather than nonradiative centers^54^ and (ii) the population of O_v_ in the XPS data, along with the observed PL emission intensity increases, indicates the high possibility and contribution of O_v_ in the PL emission mechanism. The Zheng group studied the effect of O_v_ and hydrogen treatment using single-particle PL spectroscopy.^37^ They visualized spatially distributed defects, in which higher O_v_ tends to be generated at {010} facets and contributes to PL emission.

We conducted time-correlated single photon counting (TCSPC) on undoped and doped BiVO_4_ ensemble particles using a 480 nm pulsed laser (Figure S10). TCSPC revealed the fast nonradiative decay (τ_1_) component and slower emission components (τ_2_ and τ_3_). More importantly, τ_average,int_ represents faster decay of N:BiVO_4_ (2.08 ± 0.05) and P:N:BiVO_4_ (1.22 ± 0.02) relative to BiVO_4_ (3.00 ± 0.02) and P:BiVO_4_ (2.37 ± 0.09) (Table S2).

The truncated pyramidal morphology of the BiVO_4_ crystals provides an opportunity to correlate the morphological features with PL patterns. To achieve that, three distinct dissect regions were defined as follows: an intersection between the corner of the {110} facet and two {010} facets, corner edges (CE, red areas, Figure 4A), (the remaining) intersection between the {110} and {010} facets, top edges (TE, cyan areas, Figure 4A), and an intersection between two {010} facets, side edges (SE, purple areas, Figure 4A). After the CFM and SEM images were registered (see the Supporting Information), the particle boundary dimensions were transferred to the CFM image to guide the dissection areas. The dissection areas’ mean PL intensities were extracted by reading the PL intensity of the drawn box for each area.

Figure 4A represents an exemplary confocal image of a P:N:BiVO_4_ crystal, in which the particle and top facet boundary were identified according to the corresponding SEM image. This panel also displays a dissection pattern applied to all of the particles to extract the corresponding mean PL intensities. The details of the dissection are provided in the Supporting Information.

Because the TE and CE areas contain both the basal {010} (electron-rich region) and lateral {110} (hole-rich region) facets, the probability of charge recombination is higher at these junction regions rather than solely at the basal and lateral facets. In fact, this probability increases when doping agents, as well as O_v_, are introduced to the crystalline structure of BiVO_4_.

Figure 4B shows bar plots of the extracted TE (top edge, the edge of the {010} and {110} facets excluding corner regions) mean PL intensity for BiVO_4_ and doped materials. There are 9.2% and 11.9% PL intensity enhancements for P:BiVO_4_ and N:BiVO_4_ relative to BiVO_4_, which reach 22.6% for P:N:BiVO_4_. A similar pattern was observed in the CE mean PL intensity, where N:BiVO_4_ and P:N:BiVO_4_ reveal 6.3% and 8.4% PL intensity enhancements (Figure 4C). The intensity difference reaches 20.4% when both P and N are introduced to the BiVO_4_ structure. In fact, the slight PL quenching at CE regions could be attributed to charge separation or nonradiative recombination rather than radiative recombination.

Interestingly, a major PL boost was observed at the SE, with mean PL intensities of P:BiVO_4_, N:BiVO_4_, and P:N:BiVO_4_ crystals, where, on average, there are 18.9%, 23.0%, and 31.1% increases in the PL intensity of particles (Figure 4D). This could be associated with the fact that, at the side edges, both O_v_ and elemental replacement, i.e., O with P and N, could be more probable than other structural features on the particles.

Overall, the trend of the PL emission intensity follows CE > TE > SE, which could be associated with the presence of a higher density of O_v_ defects and doping elements at various regions where the basal facets and two laterals join. Conveying an emission pattern of crystals in which the highest amount of fluorescence emission originates from the {010} and {110} intersections, as seen in Figure 4A. It is worth highlighting that the basal {110} facet is enriched with VO_4_ tetrahedral units. Because phosphorus forms PO_4_ units that preferentially occupy the same sites as VO_4_,^29^ P and N are expected to exert a greater influence on basal rather than lateral facets. This can lead to a more electron-rich surface environment, thus increasing electron–hole recombination.

Previous studies have shown that the particle size of BiVO_4_ significantly affects its performance in photocatalytic and photoelectrocatalytic water splitting.^55^ This concept can be extended to the ratio of lateral-to-basal facets, which has been identified as a key structural parameter in several recent studies.^44,51^ In our work, the squareness (SQ), which reflects the morphological shape, reveals a clear trend: as shown in Figure S11, the SQ distribution evolves from multiple populations in BiVO_4_, P:BiVO_4_, and N:BiVO_4_ to a single, near-normal distribution upon codoping.

Figure 5 A shows scatter plots that include 144 particles per material, including undoped BiVO_4_, P:BiVO_4_, N:BiVO_4_, and P:N:BiVO_4_. The statistical tests, including a regression test and full population multiple comparison results (see the Supporting Information), support the significant differences between the PL intensities. To further clarify intensity vs volume of particle trends, the scatter plot data points were averaged (Figure 5B). The PL intensity of BiVO_4_, P:BiVO_4_, and N:BiVO_4_ decreases when the particle volume increases; however, the PL intensity of P:N:BiVO_4_ reveals an independent trend with the particle size. This reveals that the solid-state PL intensity size dependency can be affected by the doping elements, where codoping eliminates the PL emission dependency on the particle volume. In other words, semiconductor doping could be an effective strategy to reduce or eliminate the effect of particle-to-particle heterogeneity among particulate semiconductor materials. One major reason could be improving the particle quality via doping, and specifically codoping; however, this hypothesis remains to be evaluated within the semiconductor community.

**Figure 5:**
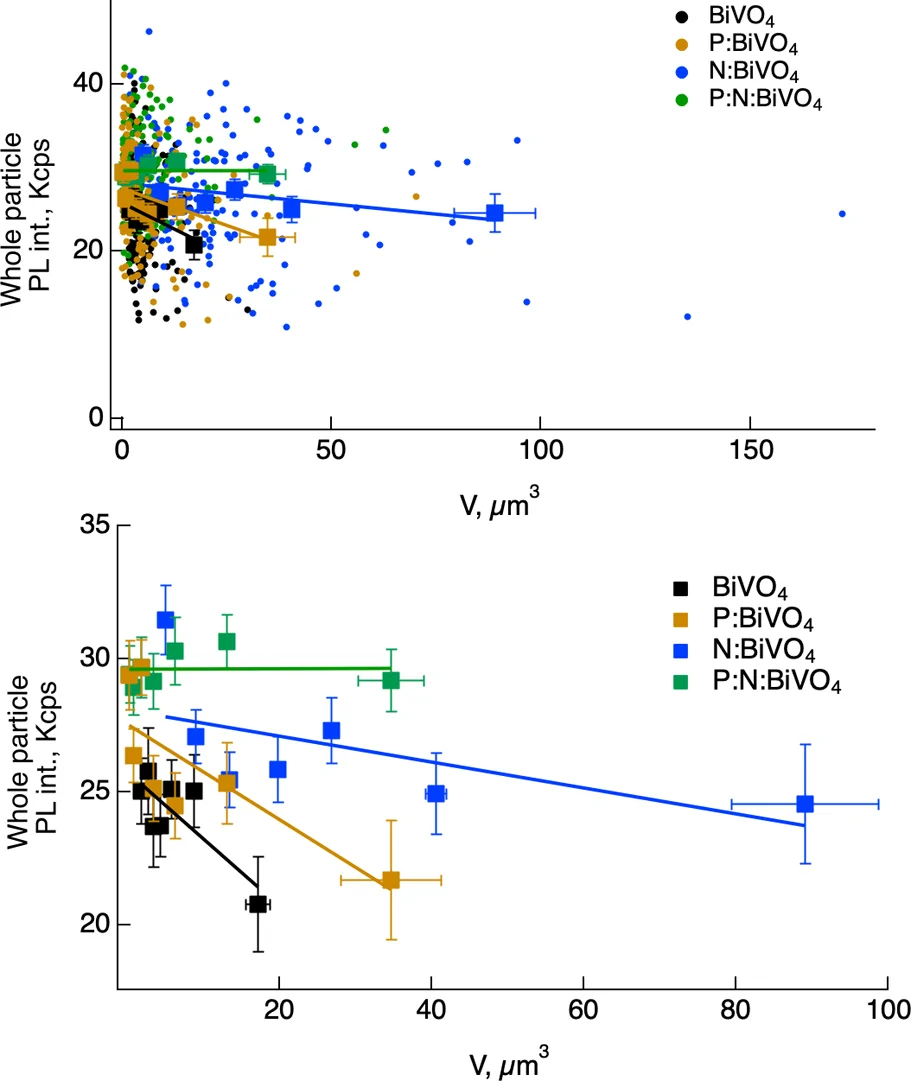
Effect of the particle volume on the PL intensity of single BiVO_4_ particles. (A) Overlaid data points and averaged binned (every 20 data points) to get general trends (symbols) of the whole-crystal PL intensity. *x* and *y* error bars are the standard deviation. (B) Zoomed-in binned plots for further clarification of the data shown in panel A. The color representations are BiVO_4_ (black), P:BiVO_4_ (brown), N:BiVO_4_ (blue), and P:N:BiVO_4_ (green). The errors are the standard deviation of the mean. The solid lines are not fits and are for further visualization of the trend for each material.

In summary, we extended the capabilities of confocal fluorescence microscopy to interrogate the photophysical properties of a series of BiVO_4_ truncated bipyramidal crystals, including undoped BiVO_4_ and chemically doped, e.g., two monodoped, P:BiVO_4_ and N:BiVO_4_, and codoped, P:N:BiVO_4_, at the single-particle level. The solid-state PL emission pattern and intensity were shown to be influenced by the dopants: the presence of P and N boosts the intensity, while the copresence of P and N not only enhances the intensity but also alters the emission pattern. The observed emission signatures were attributed to soft doping agents and to the contribution of O_v_ created within the crystal {010} and {110} facets. The truncated bipyramidal morphology of the crystals allows us to extract and investigate the physical and chemical properties of individual crystals. The dissected data revealed that the effect of doping and the creation of O_v_ is more dominant on the edges of {010} and {110} and at the junctions of two {110} facets. Nitrogen and phosphorus codoping effectively decouples charge separation and recombination dynamics from the particle size and reduces the nonradiative losses. Doping and codoping could suppress the volume-dependent behavior, with the recombination mechanism further tunable through selection of these earth-abundant dopants.

These findings highlight the importance and influence of soft chemical doping on semiconductor crystals. In addition, the photophysics of such semiconductive materials can lead to understanding the photocatalytic and photoelectrocatalytic performances as well as be used for solid-state sensors.

## Supplementary Material



## Notes

Synthesis of BiVO_4_ particles; SEM images, high-resolution XPS spectra, and EDX elemental maps; particle height and volume calculations; confocal and laser intensity parameters; CFM and SEM registration; TCSPC results; statistical study; BiVO_4_ particle SQ (PDF)

## References

[1] Hisatomi T., Yamada T., Nishiyama H., Takata T., Domen K. (2025). Materials
and systems for large-scale photocatalytic water splitting. Nat. Rev. Mater..

[2] Han S., Yang X., Zhu Y., Tan C., Zhang X., Chen J., Huang Y., Chen B., Luo Z., Ma Q. (2017). Synthesis of WOn-WX_2_ (n = 2.7, 2.9;
X =
S, Se) Heterostructures for Highly Efficient Green Quantum Dot Light-Emitting
Diodes. Angew. Chem., Int. Ed..

[3] Heckel S., Wittmann M., Reid M., Villa K., Simmchen J. (2024). An Account
on BiVO_4_ as Photocatalytic Active Matter. Acc. Mater. Res..

[4] Zhu W., Shen M., Fan G., Yang A., Meyer J. R., Ou Y., Yin B., Fortner J., Foston M., Li Z. (2018). Facet-Dependent
Enhancement in the Activity of Bismuth Vanadate Microcrystals
for the Photocatalytic Conversion of Methane to Methanol. ACS Appl. Nano Mater..

[5] Shi A., Xie P., Wang Y., Qiu Y. (2025). Photoelectrocatalytic Cl-mediated
C­(sp^3^)–H aminomethylation of hydrocarbons by BiVO_4_ photoanodes. Nat. Commun..

[6] Li C., Fan W., Chen S., Zhang F. (2022). Effective Charge Carrier Utilization
of BiVO_4_ for Solar Overall Water Splitting. Chem. Eur. J..

[7] Yan P., Jiang D., Li H., Bao J., Xu L., Qian J., Chen C., Xia J. (2019). BiPO_4_ nanocrystal/BiOCl
nanosheet heterojunction as the basis for a photoelectrochemical 4-chlorophenol
sensor. Sen. Act. B: Chem..

[8] Park Y., McDonald K. J., Choi K.-S. (2013). Progress
in bismuth vanadate photoanodes
for use in solar water oxidation. Chem. Soc.
Rev..

[9] Navarrete-Cevallos L. E., Vargas R., Espinoza-Montero P. J. (2025). Fundamentals and environmental applications
of bismuth vanadate through photoelectrocatalysis. npj Clean Water.

[10] A M., J M., Ashokkumar M., Arunachalam P. (2018). A review on
BiVO_4_ photocatalyst:
Activity enhancement methods for solar photocatalytic applications. Appl. Catal. A: Gen.

[11] Ye H., Lee J., Jang J. S., Bard A. J. (2010). Rapid Screening
of BiVO_4_-Based Photocatalysts by Scanning Electrochemical
Microscopy (SECM)
and Studies of Their Photoelectrochemical Properties. J. Phys. Chem. C.

[12] Butler K. T., Dringoli B. J., Zhou L., Rao P. M., Walsh A., Titova L. V. (2016). Ultrafast carrier
dynamics in BiVO_4_ thin
film photoanode material: interplay between free carriers, trapped
carriers and low-frequency lattice vibrations. J. Mater. Chem. A.

[13] Kim T. W., Choi K.-S. (2014). Nanoporous BiVO_4_ Photoanodes with Dual-Layer
Oxygen Evolution Catalysts for Solar Water Splitting. Science.

[14] Iwase A., Kudo A. (2010). Photoelectrochemical
water splitting using visible-light-responsive
BiVO_4_ fine particles prepared in an aqueous acetic acid
solution. J. Mater. Chem..

[15] Wang D., Li R., Zhu J., Shi J., Han J., Zong X., Li C. (2012). Photocatalytic Water
Oxidation on BiVO_4_ with the Electrocatalyst
as an Oxidation Cocatalyst: Essential Relations between Electrocatalyst
and Photocatalyst. J. Phys. Chem. C.

[16] Li R., Zhang F., Wang D., Yang J., Li M., Zhu J., Zhou X., Han H., Li C. (2013). Spatial separation
of photogenerated electrons and holes among {010} and {110} crystal
facets of BiVO_4_. Nat. Commun..

[17] Zhao Y., Ding C., Zhu J., Qin W., Tao X., Fan F., Li R., Li C. (2020). A Hydrogen
Farm Strategy for Scalable
Solar Hydrogen Production with Particulate Photocatalysts. Angew. Chem., Int. Ed..

[18] Tayebi M., Lee B.-K. (2019). Recent advances
in BiVO_4_ semiconductor materials
for hydrogen production using photoelectrochemical water splitting. Renew. Sus. Energy Rev..

[19] Lee D. K., Choi K.-S. (2018). Enhancing long-term photostability
of BiVO_4_ photoanodes for solar water splitting by tuning
electrolyte composition. Nat. Energy.

[20] Seo D., Somjit V., Wi D. H., Galli G., Choi K.-S. (2025). p-Type
BiVO_4_ for Solar O_2_ Reduction to H_2_O_2_. J. Am. Chem. Soc..

[21] Regmi C., Kshetri Y. K., Kim T.-H., Pandey R. P., Lee S. W. (2017). Visible-light-induced
Fe-doped BiVO_4_ photocatalyst for contaminated water treatment. Mol. Catal..

[22] Peng Y., Jia M., Cai J., Wang Y., Zhang Q., Gao H., Xu L., Song X., Jiang W., Zhang T. (2026). Nickel-doped BiVO_4_ induced
lattice compression and charge self-compensation:
A quantitative study on photodegradation kinetics of rhodamine B. Phys. B: Condens. Matter.

[23] Rodrigues B. S., Branco C. M., Domingos L. R. d. M., Gaubeur I., Wang Z., Chen C., Sardela M. R., Souza J. d. S. (2024). Microwave-assisted
reflux synthesis of Tungsten-doped BiVO_4_ for improved photocatalytic
activity. J. Am. Ceram. Soc..

[24] Rohloff M., Anke B., Kasian O., Zhang S., Lerch M., Scheu C., Fischer A. (2019). Enhanced Photoelectrochemical
Water
Oxidation Performance by Fluorine Incorporation in BiVO_4_ and Mo: BiVO_4_ Thin Film Photoanodes. ACS Appl. Mater. Interfaces.

[25] Baek J. H., Gill T. M., Abroshan H., Park S., Shi X., Nørskov J., Jung H. S., Siahrostami S., Zheng X. (2019). Selective and Efficient
Gd-Doped BiVO_4_ Photoanode for
Two-Electron Water Oxidation to H_2_O_2_. ACS Energy Lett..

[26] Wang S., Li C., Qi Y., Zhang J., Wang N., Liu M., Zhang B., Cai X., Zhang H., Wei S.-h. (2025). Etched BiVO_4_ photocatalyst with charge separation efficiency
exceeding 90%. Nat. Commun..

[27] Kim T. W., Ping Y., Galli G. A., Choi K.-S. (2015). Simultaneous enhancements
in photon absorption and charge transport of bismuth vanadate photoanodes
for solar water splitting. Nat. Commun..

[28] Zhang K., Jin B., Park C., Cho Y., Song X., Shi X., Zhang S., Kim W., Zeng H., Park J. H. (2019). Black phosphorene
as a hole extraction layer boosting solar water splitting of oxygen
evolution catalysts. Nat. Commun..

[29] Wu H., Zhang L., Du A., Irani R., van de Krol R., Abdi F. F., Ng Y. H. (2022). Low-bias
photoelectrochemical water
splitting via mediating trap states and small polaron hopping. Nat. Commun..

[30] Qiu W., Xiao S., Ke J., Wang Z., Tang S., Zhang K., Qian W., Huang Y., Huang D., Tong Y., Yang S. (2019). Freeing the Polarons to Facilitate
Charge Transport in BiVO_4_ from Oxygen Vacancies with an
Oxidative 2D Precursor. Angew. Chem., Int. Ed..

[31] Wang S., Yuan H., Feng J., Xu L., Guo M., Huang W., Wang S. (2026). Phosphorous-Doped High-Entropy Oxides
Enabling Full Spectrum Utilization of BiVO_4_ Photoanodes
for Efficient Water Oxidation. Adv. Funct. Mater..

[32] Mao X., Chen P. (2022). Inter-facet
junction effects on particulate photoelectrodes. Nat. Mater..

[33] Liang Q., Ouhbi H., Österbacka N., Ambrosio F., Wiktor J. (2025). Defect engineering
in BiVO_4_ photoanodes: the synergistic role of nitrogen
doping and oxygen vacancy for oxygen evolution reaction. J. Phys. Energy.

[34] Fu C., Xu B., Dong L., Zhai J., Wang X., Wang D.-Y. (2021). Highly
efficient BiVO_4_ single-crystal nanosheets with dual modification:
phosphorus doping and selective Ag modification. Nanotechnology.

[35] Tachikawa T., Ochi T., Kobori Y. (2016). Crystal-Face-Dependent
Charge Dynamics
on a BiVO_4_ Photocatalyst Revealed by Single-Particle Spectroelectrochemistry. ACS Catal..

[36] Mao J., Gu Z., Yu Y., Liu H., Qu J., An X. (2021). Revealing
Surface Charge Population on Flake-Like BiVO_4_ Photocatalysts
by Single Particle Imaging Spectroscopies. ACS
Appl. Energy Mater..

[37] Zhang Y., Liu Y., Zhang T., Gong X., Wang Z., Liu Y., Wang P., Cheng H., Dai Y., Huang B., Zheng Z. (2023). In Situ Monitoring
of the Spatial Distribution of Oxygen Vacancies
and Enhanced Photocatalytic Performance at the Single-Particle Level. Nano Lett..

[38] Li B., Lv M., Zhang Y., Gong X., Lou Z., Wang Z., Liu Y., Wang P., Cheng H., Dai Y. (2024). Single-Particle
Imaging Photoinduced Charge Transfer of Ferroelectric Polarized Heterostructures
for Photocatalysis. ACS Nano.

[39] Li B., Tong F., Lv M., Wang Z., Liu Y., Wang P., Cheng H., Dai Y., Zheng Z., Huang B. (2022). In Situ Monitoring Charge Transfer
on Topotactic Epitaxial Heterointerface
for Tetracycline Degradation at the Single-Particle Level. ACS Catal..

[40] Elbanna O., Fujitsuka M., Kim S., Majima T. (2018). Charge Carrier Dynamics
in TiO_2_ Mesocrystals with Oxygen Vacancies for Photocatalytic
Hydrogen Generation under Solar Light Irradiation. J. Phys. Chem. C.

[41] Wang W.-K., Chen J.-J., Lou Z.-Z., Kim S., Fujitsuka M., Yu H.-Q., Majima T. (2019). Single-molecule and
-particle probing
crystal edge/corner as highly efficient photocatalytic sites on a
single TiO_2_ particle. Proc. Natl.
Acad. Sci. U.S.A..

[42] Zhang Y., Lu D., Gao M., Lai M., Lin J., Lei T., Lin Z., Quan L. N., Yang P. (2019). Quantitative imaging of anion exchange
kinetics in halide perovskites. Proc. Natl.
Acad. Sci. U.S.A..

[43] Jiang Y., Le H. K. D., Verbitsky L., DeVyldere H., Oddo A. M., Pan B., Song H.-g., Zhu C., Zhu H., Scott M. C., Yang P. (2025). Emissive Chalcogenide Perovskite
Nanowires. Nano Lett..

[44] Mao X., Liu C., Hesari M., Zou N., Chen P. (2019). Super-resolution imaging
of non-fluorescent reactions via competition. Nat. Chem..

[45] Irani R., Ahmet I. Y., Jang J.-W., Berglund S. P., Plate P., Höhn C., Böttger R., Schmitt S. W., Dubourdieu C., Lardhi S. (2020). Nature
of Nitrogen Incorporation in BiVO_4_ Photoanodes through
Chemical and Physical Methods. Solar RRL.

[46] Yang J., Deng C., Lei Y., Duan M., Yang Y., Chen X., Yang S., Li J., Sheng H., Shi W., Chen C., Zhao J. (2025). Fe–N
Co-Doped BiVO_4_ Photoanode with Record Photocurrent for
Water Oxidation. Angew. Chem., Int. Ed..

[47] Wang S., Chen P., Yun J. H., Hu Y., Wang L. (2017). An Electrochemically
Treated BiVO_4_ Photoanode for Efficient Photoelectrochemical
Water Splitting. Angew. Chem., Int. Ed..

[48] Cheng C., Fang Q., Fernandez-Alberti S., Long R. (2021). Controlling Charge
Carrier Trapping and Recombination in BiVO_4_ with the Oxygen
Vacancy Oxidation State. J. Phys. Chem. Lett..

[49] Chauhan S., Singh A., Saini R., Thirumalaisamy L., Ramakrishnan J., Arul Kumaravelu T., Rajalingam T., Dong C.-L., Kandasami A. (2025). Regulation of oxygen vacancies by
gamma-irradiation in BiVO_4_ thin films and their electrochemical
performance. Ceram. Int..

[50] Zhang Y., Liu Y., Gong X., Wang Z., Liu Y., Wang P., Cheng H., Huang B., Zheng Z. (2024). Construction of piezoelectric
photocatalyst Au/BiVO_4_ for efficient degradation of tetracycline
and studied at single-particle level. Chem.
Synth..

[51] Banik A., Maekawa H., Fajardo J., Zutter B., Alcorn F. M., Kumar S., Watanabe K., Kudo A., Ge N.-H., Talin A. A., Sambur J. B. (2025). Unequal {110} Facets: The Potential
Role of Intraparticle Heterogeneity and Facet Termination in Photoelectrochemical
Activity of Single BiVO_4_ Particles. ACS Nano.

[52] Su S., Siretanu I., van den Ende D., Mei B., Mul G., Mugele F. (2024). Nanometer-Resolved Operando Photo-Response of Faceted
BiVO_4_ Semiconductor Nanoparticles. J. Am. Chem. Soc..

[53] El
Ouardi O., Ladib H., Vigolo B., Alami J., Makha M. (2025). Unveiling the role of doping and intrinsic vacancies in BiVO_4_ for enhanced photoelectrochemical water splitting: a first-principles
study. Int. J. Hydrog. Energy.

[54] Wang W., Strohbeen P. J., Lee D., Zhou C., Kawasaki J. K., Choi K.-S., Liu M., Galli G. (2020). The role of surface
oxygen vacancies in BiVO_4_. Chem.
Mater..

[55] Tan H. L., Amal R., Ng Y. H. (2016). Exploring the Different
Roles of
Particle Size in Photoelectrochemical and Photocatalytic Water Oxidation
on BiVO_4_. ACS Appl. Mater. Interfaces.

