# Supplementary material for: Single-Particle View of Doping-Controlled Charge Recombination in BiVO4
Source: Nano Lett. 2026 Oct 1;26(40):13887–95. doi: 10.1021/acs.nanolett.6c03155 (PMC13674328; doi:10.1021/acs.nanolett.6c03155)
Supplement: Supplementary file 1 [file nl6c03155_si_001.pdf]

Single-Particle View of Doping-Controlled Charge Recombination in $BiVO_4$

*Sean McCoy,[1] Devon Stritesky,[1,2] Liam Todd,[3] Marissa Ashworth[1] and Mahdi Hesari[1]**

[1]Department of Chemistry, State University of New York at Oswego, Oswego, New York, 13126, United States.

[2]Department of Computer Science and Engineering, State University of New York at Oswego, Oswego, New York, 13126, United States.

[3]Department of Atmospheric and Geological Sciences, State University of New York at Oswego, Oswego, New York, 13126, United States.

*Email: mahdi.hesari@oswego.edu

Table of contents

Chemicals S3
Instrument S3
Synthesis of bismuth vanadate particles S4
Calculation of the particle height and volume S7
Confocal fluorescence microscopy S8
Registration of the CFM and SEM images S11
Time-correlated single-photon counting S14
Statistical investigation of the doping effect on PL S15
Correlation between $BiVO_4$ particle squareness and photoluminescence intensity S16
Reference: S17

List of Figures

Figure S1. Scanning electron microscopy image of ensemble and single (inset) (A) $BiVO_4$, (B) P:$BiVO_4$, (C) N:$BiVO_4$, and (D) P:N:$BiVO_4$ particles. S5
Figure S2. Scanning electron microscopy-energy dispersive X-ray elemental map of a P:N:$BiVO_4$ particle. (A) SEM image of a P:N:$BiVO_4$ particle, (B) bismuth, (C) vanadium, (D) oxygen, (E) phosphorus, and (F) nitrogen. The scale bar is 1 μm. S6
Figure S3. Curve-fitted high-resolution XPS spectra of $BiVO_4$ crystals of (A-C) $BiVO_4$, (D-G) P:$BiVO_4$, (H-K) N:$BiVO_4$, and (L-Q) P:N:$BiVO_4$ particles. S7
Figure S4. Measured photoluminescence intensity of an exemplary $BiVO_4$ single particle at (A) 615 and (B) 740 gain. The open circles show the collected PL intensity of the background. The cyan bar highlights that at 50% laser intensity under both gain signals, no saturation is observed. S9
Figure S5. Photoluminescence stability of $BiVO_4$ single particle. PL intensity versus time of (A) P:$BiVO_4$, and (B) N:$BiVO_4$ monitored over 950 sec. S10
Figure S6. Gaussian curve-fitted histogram of annealed $BiVO_4$. S10
Figure S7. Procedure of overlaying the SEM-determined crystal structural contour onto the CFM image. (A) Wide field optical images of region of interest, (B) corresponding SEM image of the region including a zoomed-in area to highlight the intact morphology of particles at lower population, (C) CFM image of a crystal, (D) SEM image of the same particle shown in (C), (E) a crystal Images are scaled to each other with respect to their scale bars and are overlayed onto each (F) drawn outline of crystal based on SEM image, (G) outline of crystal is placed on confocal image (H) image is then used to obtain dissected fluorescence intensities of different region. . S12
Figure S8. (A) Brightfield and (B) confocal images of representative multiple single particles of N:$BiVO_4$ under the same conditions as the individual ones. S13
Figure S9. (A-C) Brightfield and (D-F) confocal images of adjacent P:$BiVO_4$ particles collected under the same conditions as the individual ones. S13
Figure S10. Normalized time-correlated single-photon counting (TCSPC) of $BiVO_4$ particles. The intensity-time traces were background-subtracted, corrected for Rayleigh scattered light, and fitted using eq. 4 with three decay constants. S14
Figure S11. Correlation between $BiVO_4$ particle squareness and photoluminescence intensity. Scatter plot and histogram of the mean whole-particle PL intensity vs. squareness of single particles of (A) $BiVO_4$, (B) P:$BiVO_4$, (C) N:$BiVO_4$, and (D) P:N:$BiVO_4$. S16

Chemicals

The following chemicals, without further purification, were used to synthesize $BiVO_4$ and P:$BiVO_4$; bismuth (III) Nitrate Pentahydrate (99.9%, Thermo Fisher), Ammonium Metavanadate (99.9%, Scientific Fisher), phosphoric acid (99.99%, Sigma-Aldrich), and nitric acid (Scientific Fisher). Nitrogen gas (99.99%) was used to obtain N:$BiVO_4$. 30% Ludox solution was purchased from Sigma-Aldrich. All reagents were used as received without further purification.

Instrument

UV-Visible spectra were acquired in transmission and diffuse reflectance of powder films of $BiVO_4$ crystals, using a Cary 5000, a UV/Vis/NIR optical spectrophotometer.

ThermoFisher Scientific Nexsa G2 Surface Analysis System, equipped with a monochromatic Al K-alpha X-ray source (1486.6 eV) and 180° hemispherical analyzer with a 128-channel detector. The measurement take-off angle is 90° relative to the sample surface, also known as "normal emission." An X-ray spot size of 400 µm and a flood gun for charge compensation were used. This gun is dual-source, low-energy Ar^+ and electrons. A standard Nexsa G2 powder well plate for all samples except for sample loading. All Survey spectra had the following: Pass Energy (eV) = 200.000, Dwell time (ms) = 10, and Energy Step Size (eV) = 1.000. All high-resolution spectra had the following: Pass Energy (eV) = 50.000, Dwell time (ms) = 50, and Energy Step Size (eV) = 0.100. The XPS high-resolution spectra were curve-fitted using CasaXPS software.[1]

Powder X-Ray diffraction (XRD) spectra were collected using a Bruker D2 Phaser with Cu Kα radiation.

Photoluminescence spectra were collected on the $BiVO_4$ materials using a Renishaw InVia Confocal Raman Microscope equipped with a 488 nm laser, a 488 nm long-pass Rayleigh filter, and a CCD camera detector (299-700 nm). The laser power was optimized to 35±2 mW, with a beam spot diameter of 0.9±0.1 µm. The spectra were accumulated for 20 seconds. Time-correlated single-photon counting (TCSPC) spectra were collected using FLS980. The solid particles were packed in a 10 mm × 0.2 mm quartz cell, and a 485 nm pulsed laser was used to excite the material.

A low-vacuum JEOL JSM-6610LV scanning electron microscope equipped with X-ray energy-dispersive spectroscopy (EDS) was used to obtain SEM images and EDX elemental analysis. The SEM images, EDX results, and maps were acquired at 30 kV and 15 kV, respectively.

The trace phosphorus percentage in P:$BiVO_4$ and P:N:$BiVO_4$ was measured based on wavelength-dispersive spectroscopy (WDS) using a Cameca SXFive electron probe microanalyzer; 15 kV accelerating voltage, 20 nA beam current, and a focused beam (1 to 2 µm in diameter), and Scandium phosphate ($ScPO_4$) was used as a standard.

The trace amount of phosphorus was measured by digestion in aqua regia and then analyzed by inductively coupled plasma optical emission spectroscopy (ICP-OES, Agilent 5800).

Synthesis of bismuth vanadate particles

$BiVO_4$ was synthesized by mixing 3 mmol of bismuth nitrate trihydrate in 30 mL of 1 M nitric acid and stirring for 10 minutes. 3 mmol of ammonium vanadate was added, resulting in a yellowish mixture, and stirred for an additional 10 minutes. The solution pH was monitored throughout the reaction. Then, the mixture pH was adjusted to 1.0 with ammonium hydroxide, yielding an opaque yellow slurry, which was stirred for 2 h before being transferred to a 50 mL Teflon hydrothermal reactor. The reaction was maintained at 80 °C for 48 h in an electric oven and then cooled naturally. The bright yellow crystals were collected using a gravity filter and extensively washed with DI water to remove unreacted starting salts and dried at room temperature and at 60 °C before analysis.

Synthesis of phosphorus-doped bismuth vanadate (P:$BiVO_4$): A procedure similar to that used for $BiVO_4$ was employed to obtain P:$BiVO_4$ crystals; however, to introduce phosphorus into the crystalline material, phosphoric acid was used. In detail, 3 mmol of Bi^{3+} was dissolved in 30 mL of nitric acid, then 0.27 mmol of ammonium vanadate and, lastly, 0.03 mmol of phosphoric acid (98%) were added to the mixture, which was stirred for 10 minutes before the pH was adjusted to 1.0. The mixture was stirred for 2 h at room temperature and then transferred into a 50 mL hydrothermal autoclave reactor. The reactor was held at 80 °C in an electric oven for 48 h, then cooled to room temperature, gravity-filtered, washed extensively with DI water, and dried.

Synthesis of nitrogen-doped bismuth vanadate (N:$BiVO_4$): To achieve N:$BiVO_4$, 100 mg of undoped $BiVO_4$ was loaded onto an aqua regia-treated quartz boat and placed at the center of an electric oven equipped with a quartz tube. A flow of 0.1 L/min of 99.999% pure nitrogen was introduced into the quartz tube. At the same time, the undoped $BiVO_4$ was annealed at 450 °C for 8 h. Subsequently, the N:$BiVO_4$ was cooled to room temperature naturally and collected for subsequent analysis.

To achieve P:N:$BiVO_4$ co-doped crystals, 100 mg of P:$BiVO_4$ was loaded onto an aqua regia-treated quartz boat and placed at the center of an electric oven equipped with a quartz tube. A flow of 0.1 L/min of 99.999% pure nitrogen was introduced into the quartz tube. At the same time, the undoped $BiVO_4$ was annealed at 450 °C for 8 h. Subsequently, the P:N:$BiVO_4$ was cooled to room temperature naturally and collected for future analysis.

Sample analyses were conducted using independently synthesized batches of each material; however, reproducibility of results depends on crystal size and on removing the residual starting materials. In addition, it is recommended to purify the materials immediately after synthesis.

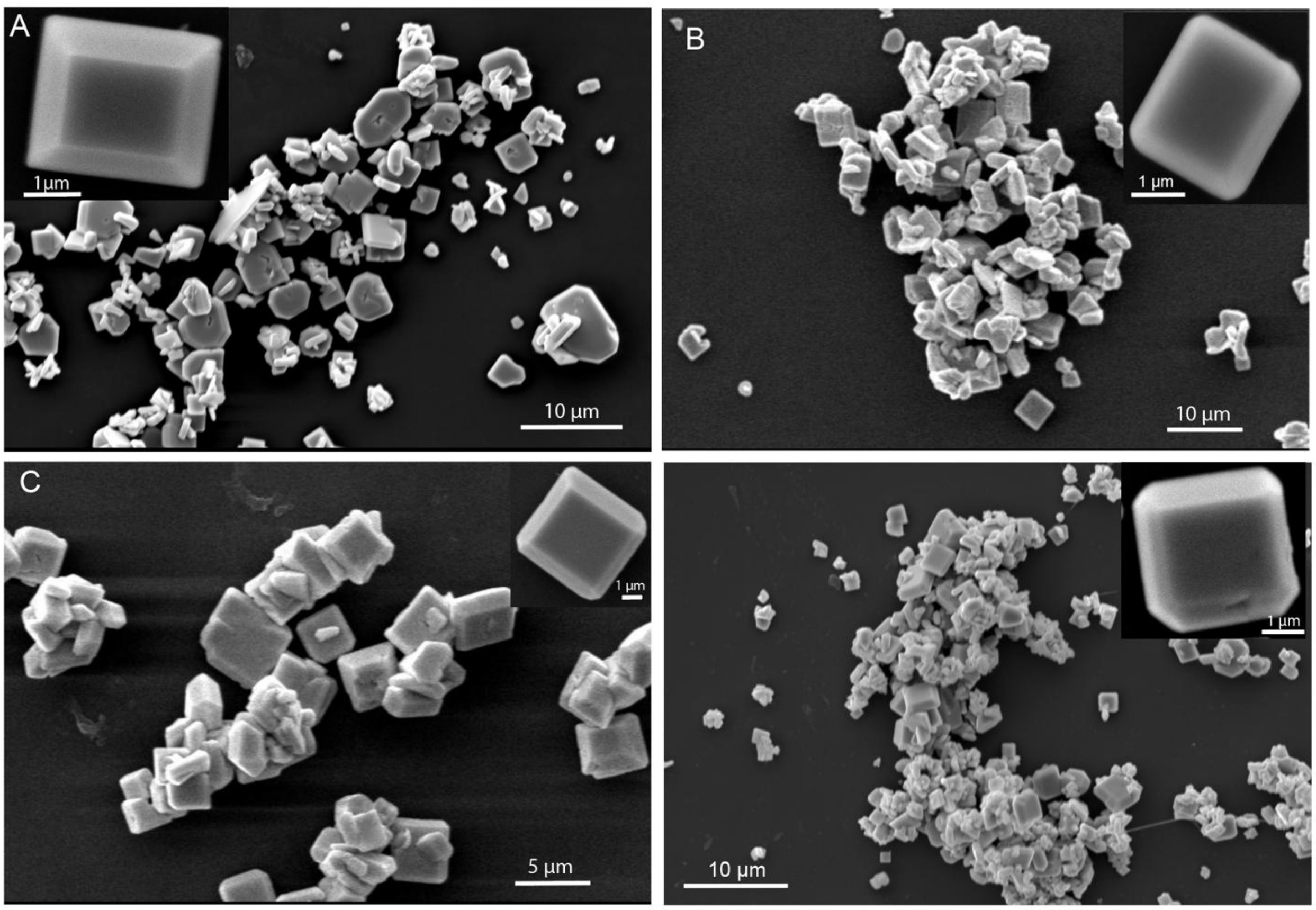

Figure S1. Scanning electron microscopy image of ensemble and single (inset) (A) $BiVO_4$, (B) P:$BiVO_4$, (C) N:$BiVO_4$, and (D) P:N:$BiVO_4$ particles.

Table S1. Elemental analysis of $BiVO_4$ materials.

	W%				Atomic %			
	$BiVO_4$	P:$BiVO_4$	N:$BiVO_4$	P:N:$BiVO_4$	$BiVO_4$	P:$BiVO_4$	N:$BiVO_4$	P:N:$BiVO_4$
**Bi**	59.9	67.3	65.5	67.5	13.1	19.3	16.9	18.8
**V**	13.5	16.1	14.7	14.9	12.1	18.9	15.5	17
**O**	26.5	16.5	19	15.8	74.9	61.7	64.3	57.3
**P**	-	0.1		0.3	-	0.2	-	0.6
**N**	-	-	0.8	1.5	-	-	3.3	6.2

The reported trace phosphorus percentage using WDS is the average of 10 independent measurements (n=10) of individual bulk and particle spots. The obtained phosphorus w% results for P:$BiVO_4$ and P:N:$BiVO_4$ are 0.125±0.062% and 0.093±0.067%. The ICP result shows P w% to be 0.083±0.02%.

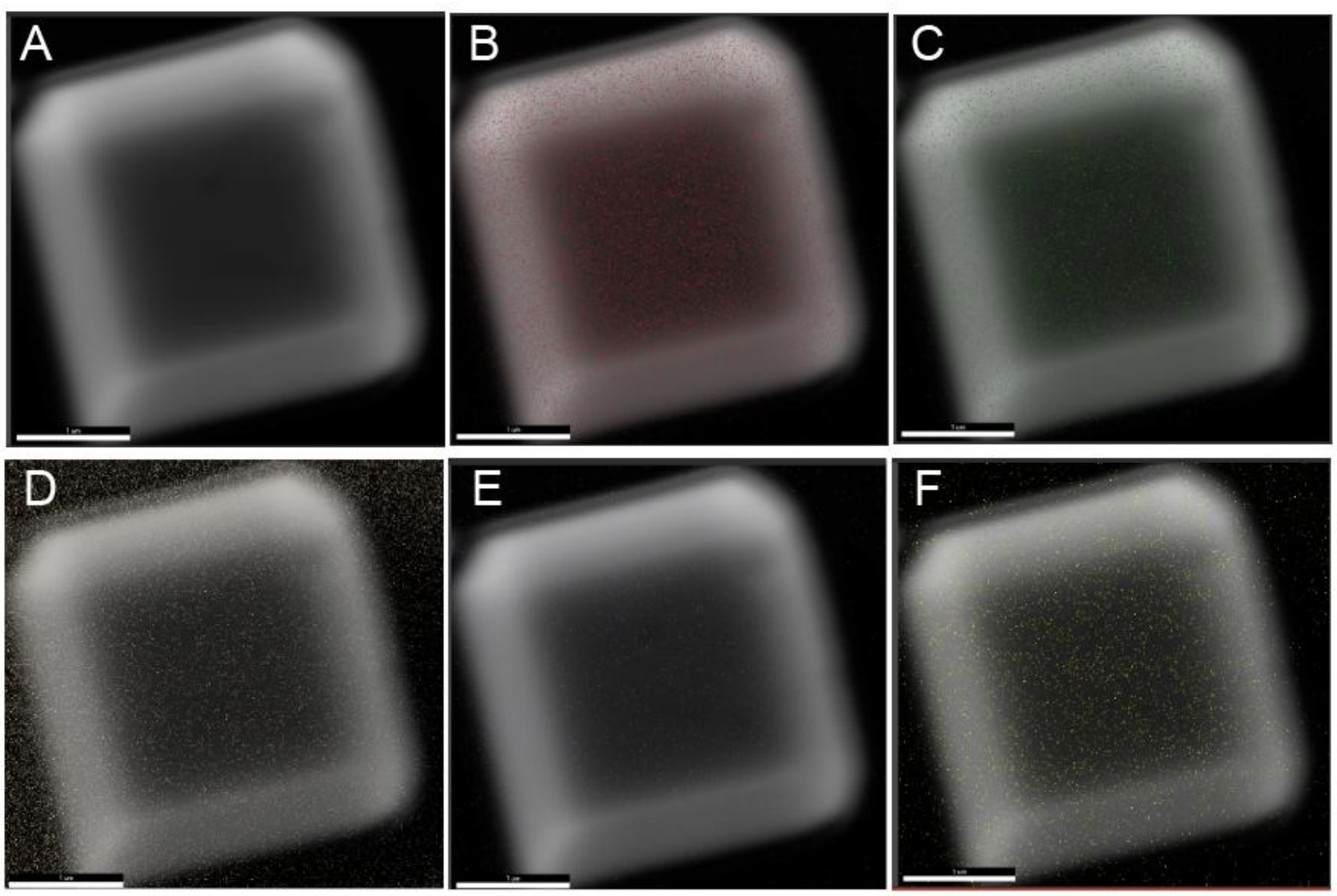

Figure S2. Scanning electron microscopy-energy-dispersive X-ray elemental map of a P:N:$BiVO_4$ particle. (A) SEM image of a P:N:$BiVO_4$ particle, (B) bismuth, (C) vanadium, (D) oxygen, (E) phosphorus, and (F) nitrogen. The scale bar is 1 μm.

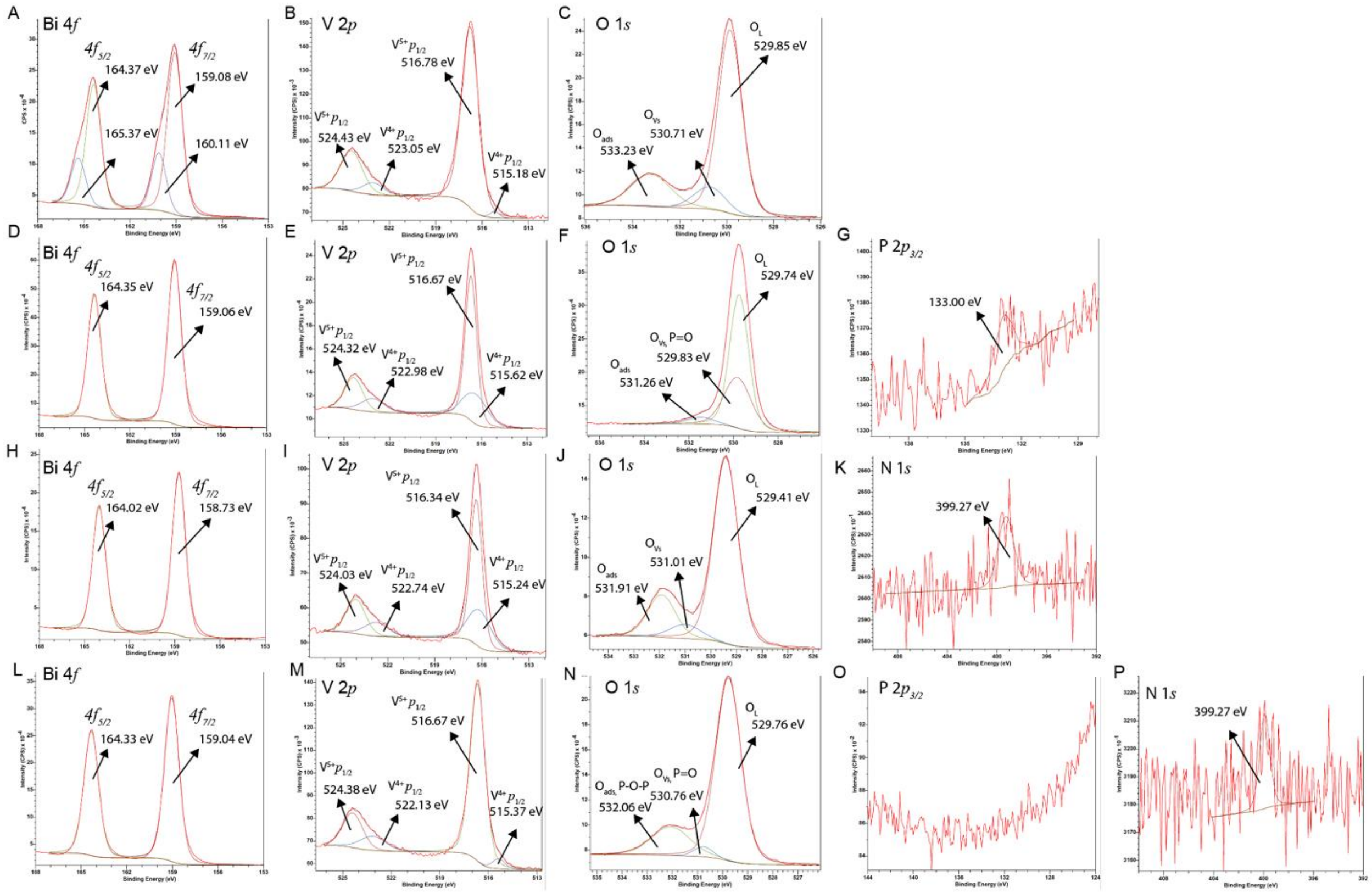

Figure S3. Curve-fitted high-resolution XPS spectra of $BiVO_4$ crystals of (A-C) $BiVO_4$, (D-G) P:$BiVO_4$, (H-K) N:$BiVO_4$, and (L-Q) P:N:$BiVO_4$ particles.

Calculation of the particle height and volume

The height of the particles was calculated using equation (1)[2] and (2), as shown below;

$$h = \frac{x-y}{\sqrt{2}} \qquad \text{eq. (1)}$$

$$H = 2h \qquad \text{eq. (2)}$$

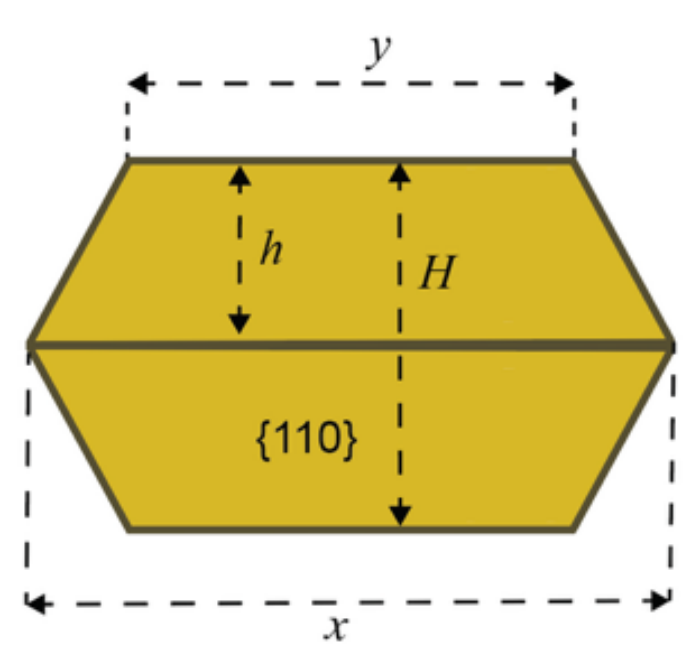

Figure 1G displays boxplots of the measured length (x) for $BiVO_4$ 2712.0 ± 629.9 nm, P:$BiVO_4$ 3155.0 ± 977.1 nm, N:$BiVO_4$ 3107 ± 892.1 nm, and P:N:$BiVO_4$ 3358 ± 855.1 nm. Figure 1G

shows the calculated height (H) of $BiVO_4$ 964.8 ± 747.7 nm, P:$BiVO_4$ 2301 ± 813.8 nm, N:$BiVO_4$ 503.9 ± 432.4 nm, and P:N:$BiVO_4$ 517.6 ± 446.9 nm.

The particle volume was estimated using eq. 3 (ref. 2):

$$V_{TB} = \frac{1}{3}h(x^2 + y^2 + xy) \quad \text{eq. (3)}$$

$$V_{particle} = 2V_{TB} \quad \text{eq. (4)}$$

where h is the estimated half of the particle height, and x and y length of the basal and lateral facets.

Confocal fluorescence microscopy

Single-particle confocal fluorescence microscopy. Figure 1E represents a confocal setup. 1 mg of the particles was dispersed in 5 mL of DI water and sonicated for 30 minutes. A 200 µL aliquot of the suspension was then drop-cast onto a cleaned glass coverslip (Fisher Scientific) and dried in a muffle furnace at 400 °C for 2 h. Next, the $BiVO_4$ was mounted on a laser scanning confocal microscope (LSM) 700. Brightfield images of the samples were acquired using 5X and 20X air objectives (Zeiss) for subsequent SEM image analysis. The confocal fluorescence images of the single particle were recorded using a 63X oil objective (Zeiss, numerical aperture 1.4). A 488 nm laser was employed to excite the single particle. The laser intensity was optimized based on the saturated photoluminescent (PL) signal (Figure S4). Confocal images were collected at a 7.26 µm × 7.26 µm frame size (i.e., pixel 0.004 µm × 0.004 µm). The recorded PL images were later overlaid on the corresponding SEM images to extract subfaces and dissected information (Figure S5).

The following optimized experimental conditions were used to collect the confocal images: a Zeiss LSM 700 confocal microscope equipped with Zen Blue for brightfield and Zen black for confocal fluorescence imaging. A 488 nm laser (10 mW) at the source and 4.75 mW/cm^2 at the objective was used to illuminate the single crystals. Typically, a 63X oil objective was used to collect the brightfield and fluorescence images of individual particles. The fluorescence signal was collected using a T-PMT (model: R5070, detection range 300-850 nm).

Images were acquired using a 16-bit range (0-65,535). Three acquisition resolutions were used depending on experimental requirements. The 2048 × 2048-pixel image resolution was used to obtain high-resolution images of individual crystals. The increase in pixels allowed for higher-quality mapping.

Digital Zoom: a digital zoom of 14X (1764X total) was used for individual crystal imaging. This field of view is 7.26×7.26 µm.

Averaging: averaging is set to 1 for individual crystal imaging, providing a quick snapshot of the crystal. Individual crystal images had a frame time of 3.87s. The PL intensity, whole particle and/or selected dissected areas, was obtained based on a raster scan mechanism and integration of intensity in the range of the T-PMT, following eq. (3):

$$PL = \int_{800}^{300} \frac{dI_{PL}}{d\lambda} \qquad \text{eq. (3)}$$

Pinhole: The pinhole is increased to 2 AU (81.9 µm) to improve the signal/noise ratio. The resolution benefits from having the pinhole at 1 AU were deemed insignificant for our samples.

Laser Intensity: For the 488 nm laser, 50% of its intensity was used, which allowed us to use less gain and obtain a better signal/noise ratio. Laser intensities higher than 50% have been observed to create a decrease in crystal intensity over time. Figure S4 shows intensity and signal-to-noise ratio on an exemplary $BiVO_4$ crystal. Gain was increased by 650, providing better observation of the PL signal per crystal without saturating the signal (Figure S4).

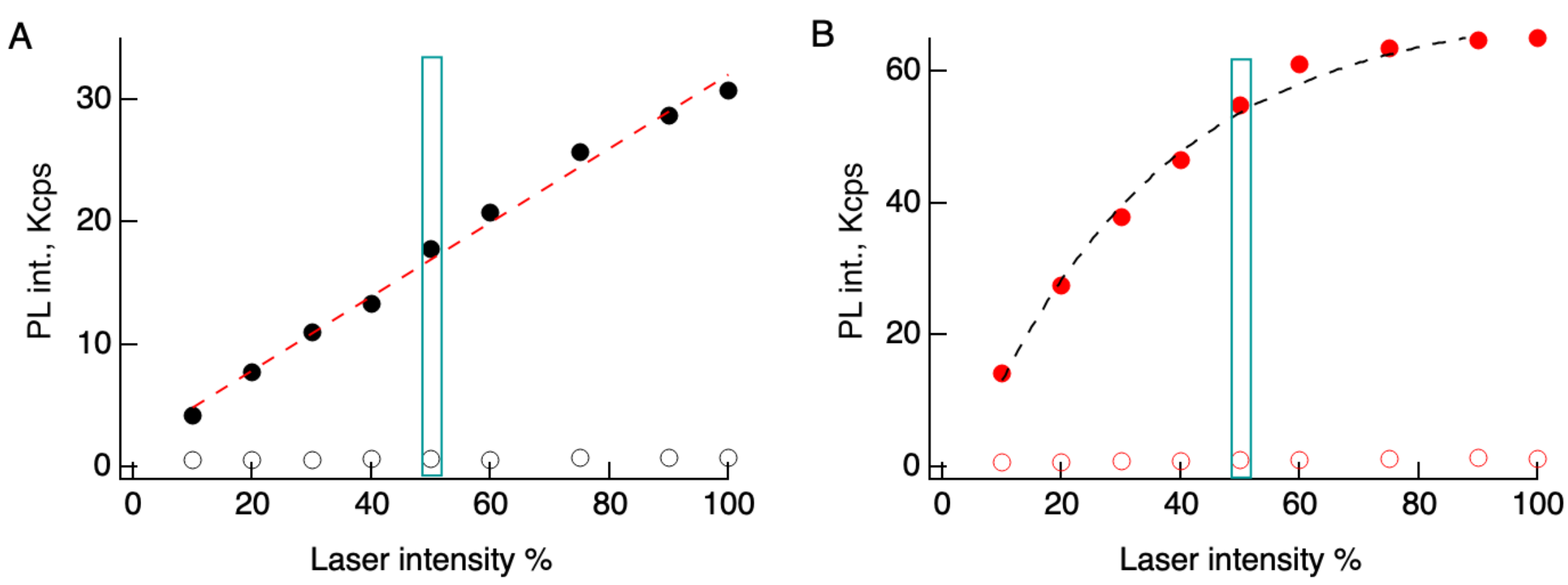

Figure S4. Measured photoluminescence intensity of an exemplary $BiVO_4$ single particle at (A) 615 and (B) 740 gain. The open circles show the collected PL intensity of the background. The cyan bar highlights that at 50% laser intensity under both gain signals, no saturation is observed.

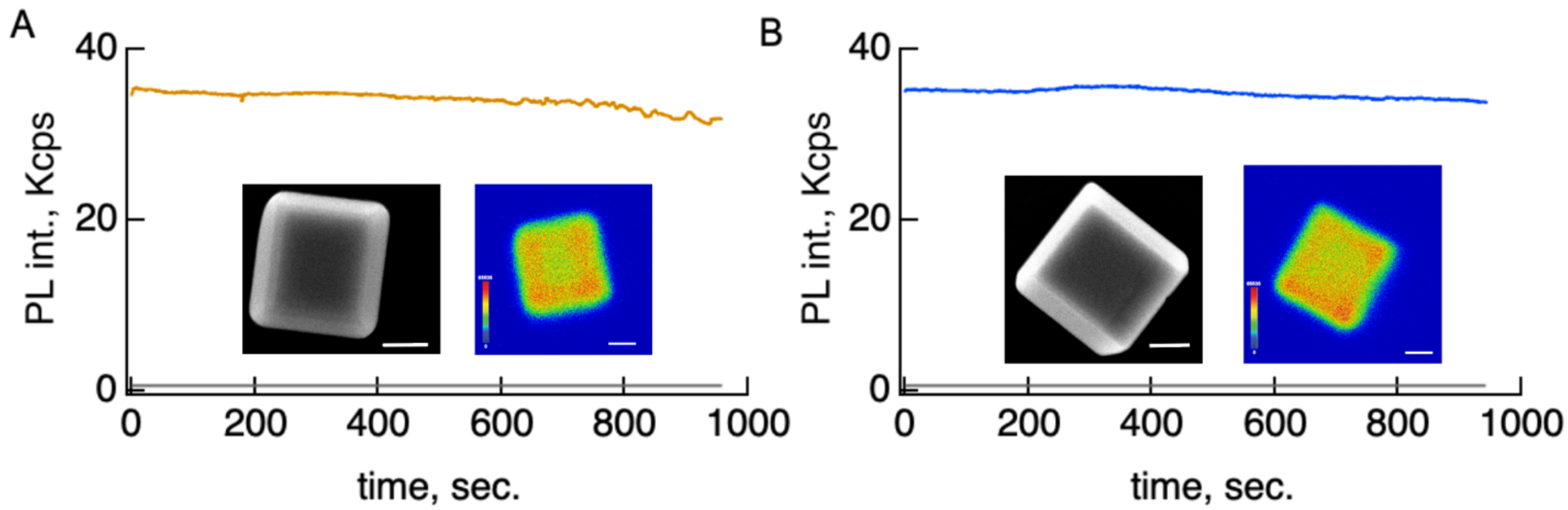

Figure S5. Photoluminescence stability of $BiVO_4$ single particle. PL intensity versus time of (A) P:$BiVO_4$, and (B) N:$BiVO_4$ monitored over 950 sec.

The PL intensity of particles was monitored for 950 seconds, which is much longer than the time required to acquire individual confocal images (e.g., 4 seconds), and no substantial intensity changes or fluctuations were observed (Figure S5). This observation supports the stability of the observed PL emission.

To confirm whether annealing temperature (e.g., 400 ºC) alters the surface chemistry and population of Ov, we specifically annealed a fresh batch of undoped $BiVO_4$, dried it at room temperature, and annealed it at 450ºC for 8 h in air. Then, confocal images of a population of 50 particles were collected under the same microscopic conditions and statistically treated as other samples. A histogram plot of the particle populations is shown in Figure S6. The results revealed a mean whole-particle intensity of 22.62 ± 4.85 Kcps.

The nitrogen content of N:$BiVO_4$ after annealing at 400 ºC for 2 h was examined using EDX. The obtained results turned out to be 0.81 w% and 16.91 atm%, which are very similar to those reported in Table S1.

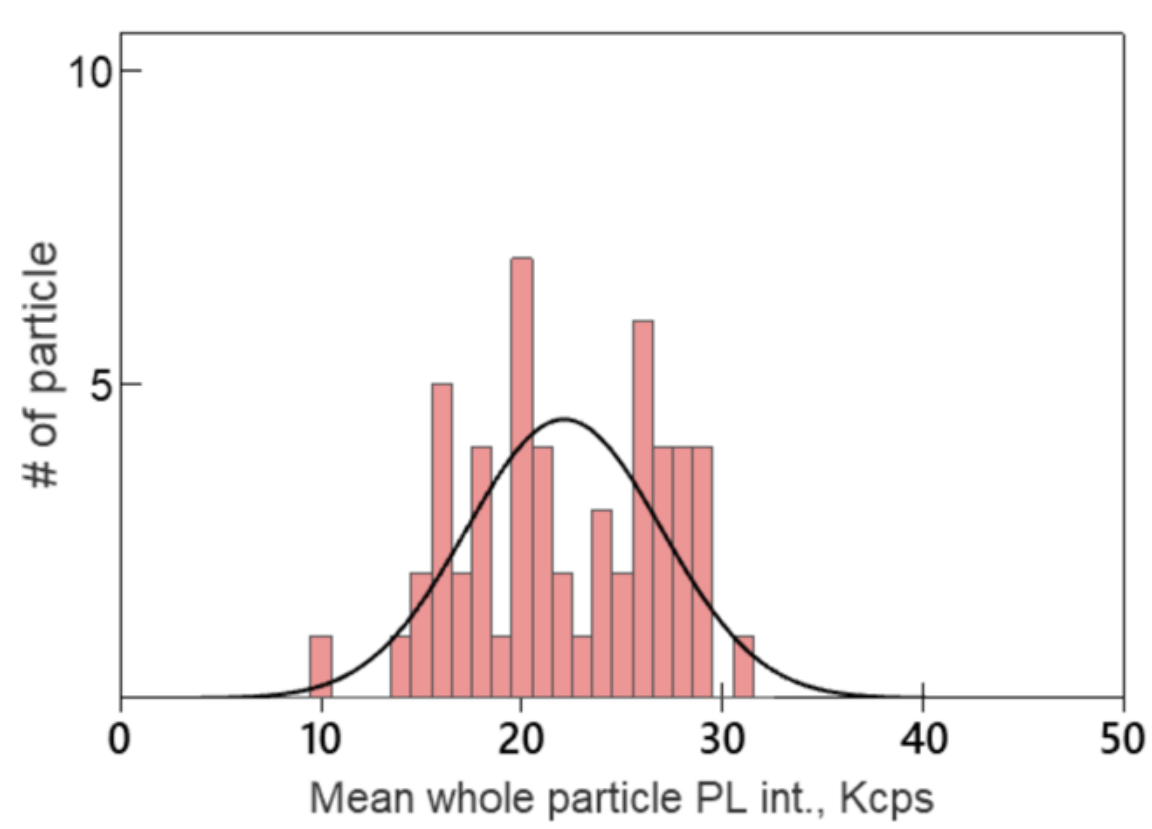

Figure S6. Gaussian curve-fitted histogram of annealed $BiVO_4$.

Registration of the CFM and SEM images

To obtain detailed structural fluorescence microscopy information, we employed SEM, which provides higher resolution of individual $BiVO_4$ particles. Figure S7A displays optical images of a wide field of view in which a cross marker was used to identify and guide the location of particles of interest. Using the digital coordinates, the SEM image of an individual particle was obtained with potentially high resolution. Next, the SEM (Figure S7C) and confocal (Figure S7C) images were scaled using the scale bar provided in each image. Subsequently, the two images were overlaid and required scaling and rotation to align them (Figure S7E). To improve the resolution of the overlapped images, the opacity of the CFM image was reduced. Next, the particle SEM image was outlined to define the particle boundary, the top face, and the joint boundaries (Figure S7F). The SEM image was removed, and the outlined particle boundary was obtained (Figure S7G) and used for dissecting the different regions over the individual $BiVO_4$ crystal as follows:

- Whole Crystal: measurement across the entire crystal
- Top facet: measurement of the {010} facet
- Corner edge (CE): measurement of the active site region located on the corner edge formed by {010} and {110} facets.
- Top Edge (TE): measurement of the edge of the {010} and {110} facets excluding corner regions
- Side Edge (SE): measurement of the edges of the {110} facet.

All confocal images yield diffraction-limited lateral and axial resolutions of ~200 nm and ~0.7–0.8 μm, respectively. The 2 Airy Unit pinhole improves signal-to-noise ratio at the cost of a slightly broader optical section (~0.8–1.2 μm). Although digital oversampling was applied, this enhances visualization smoothness only and does not improve optical resolution, which remains PSF-limited at ~200 nm laterally. Consequently, the spatial accuracy of sub-micrometric features such as facet boundaries is governed by optical resolution (~200–300 nm) rather than pixel size. For correlative confocal–SEM analysis, modality registration introduces an additional spatial uncertainty of ~100–200 nm, which is negligible at the μm scale of the features examined.

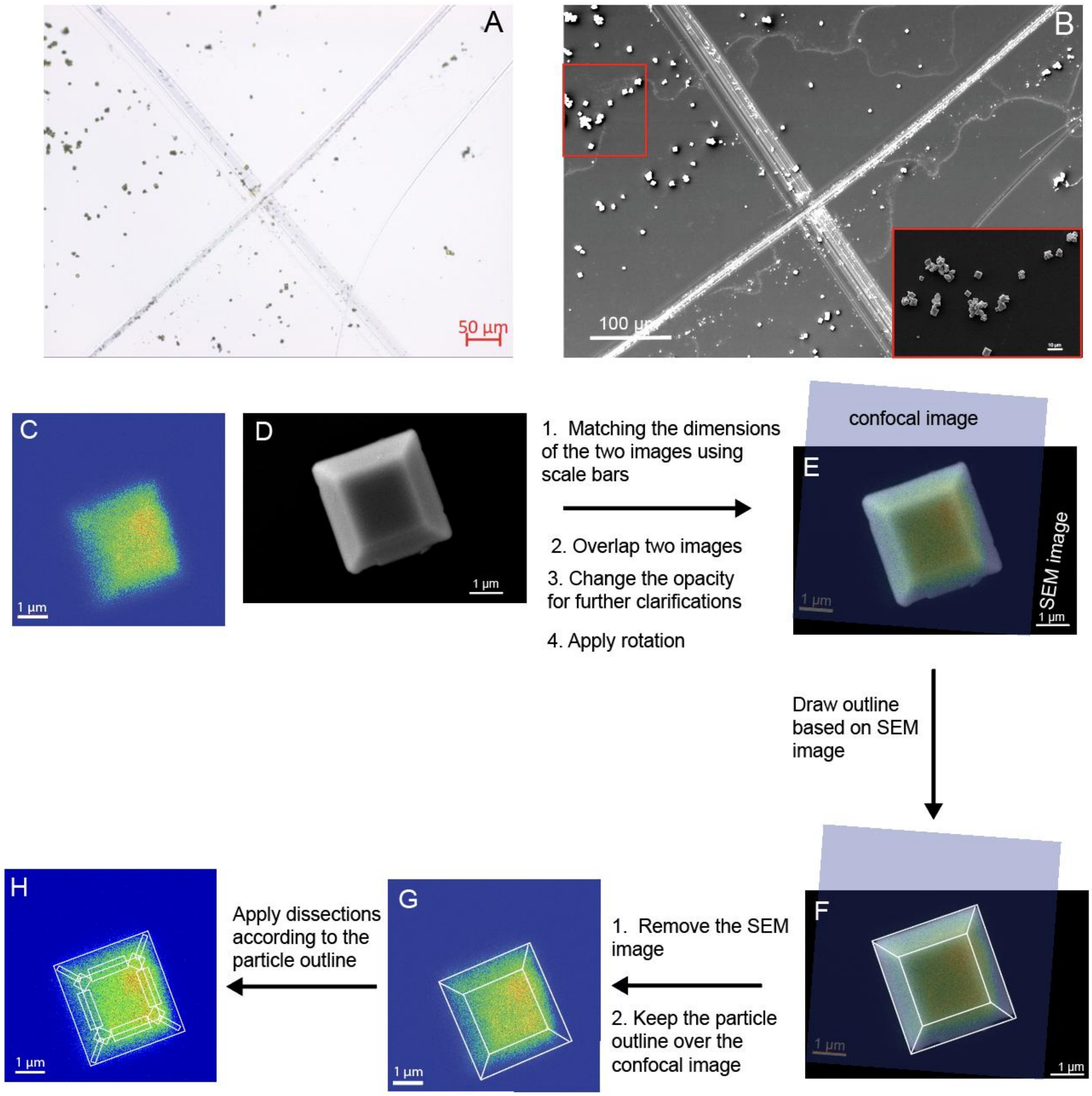

Figure S7. Procedure of overlaying the SEM-determined crystal structural contour onto the CFM image. (A) Wide field optical images of region of interest, (B) corresponding SEM image of the region including a zoomed-in area to highlight the intact morphology of particles at lower population, (C) CFM image of a crystal, (D) SEM image of the same particle shown in (C), (E) a crystal Images are scaled to each other with respect to their scale bars and are overlayed onto each (F) drawn outline of crystal based on SEM image, (G) outline of crystal is placed on confocal image (H) image is then used to obtain dissected fluorescence intensities of different region.

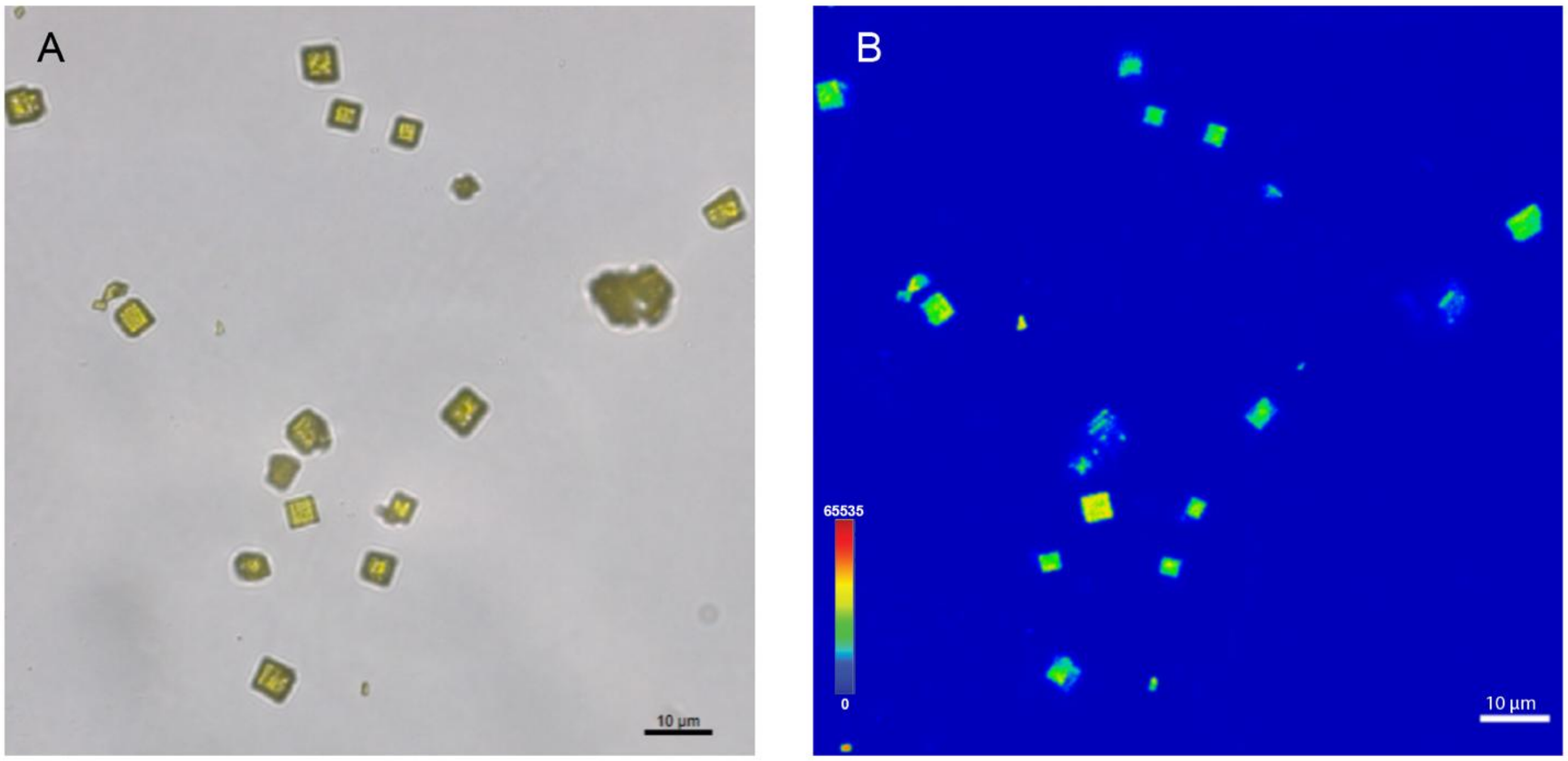

Figure S8. (A) Brightfield and (B) confocal images of representative multiple single particles of N:$BiVO_4$ under the same conditions as the individual ones.

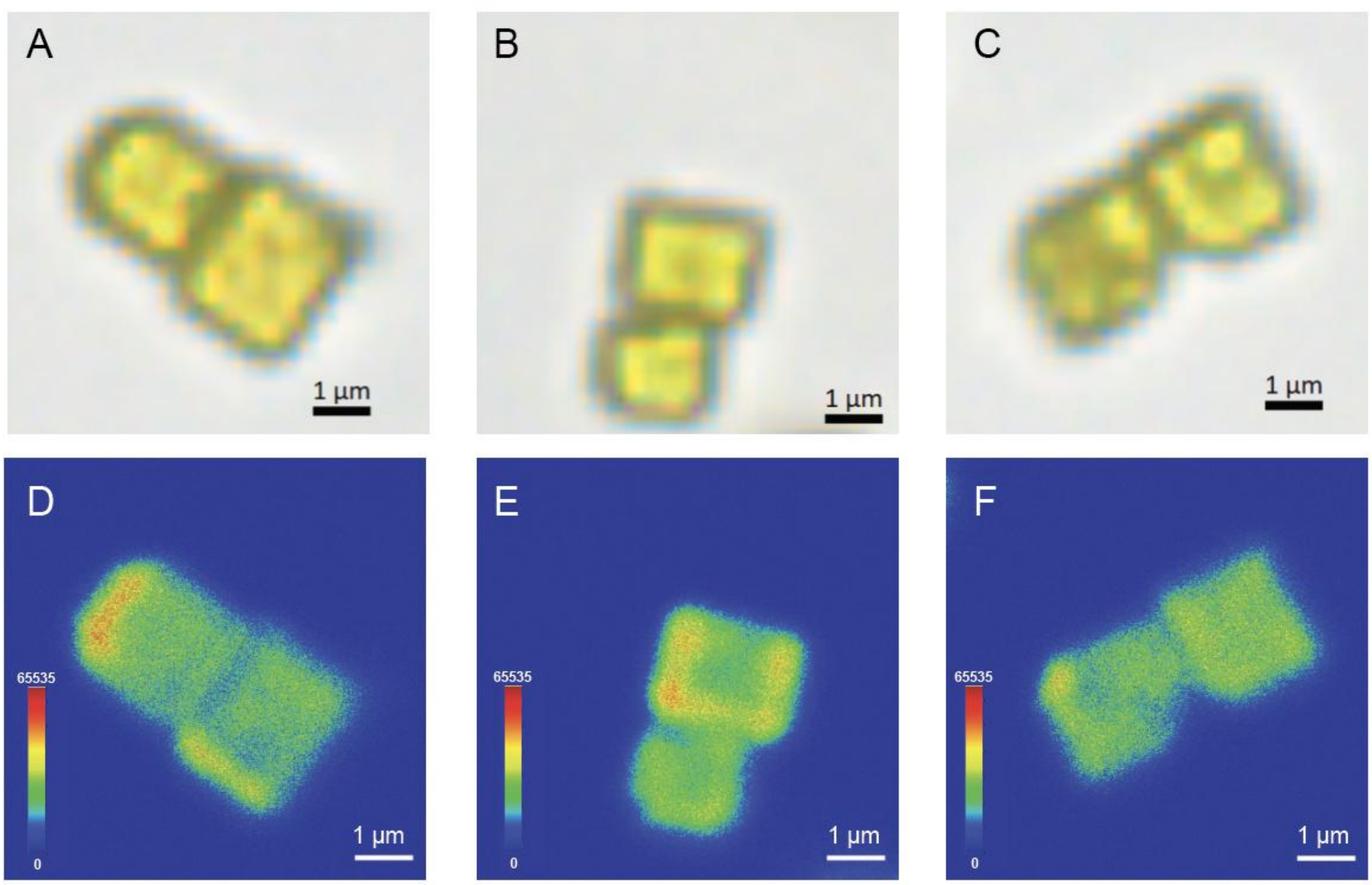

Figure S9. (A-C) Brightfield and (D-F) confocal images of adjacent P:$BiVO_4$ particles collected under the same conditions as the individual ones.

Time-correlated single-photon counting

We conducted time-correlated single-photon counting (TCSPC) of ensemble $BiVO_4$ particles (i.e., undoped and doped) using a 480 nm pulse laser. Figure S10 displays decay traces obtained for $BiVO_4$, P:$BiVO_4$, N:$BiVO_4$, and P:N:$BiVO_4$.

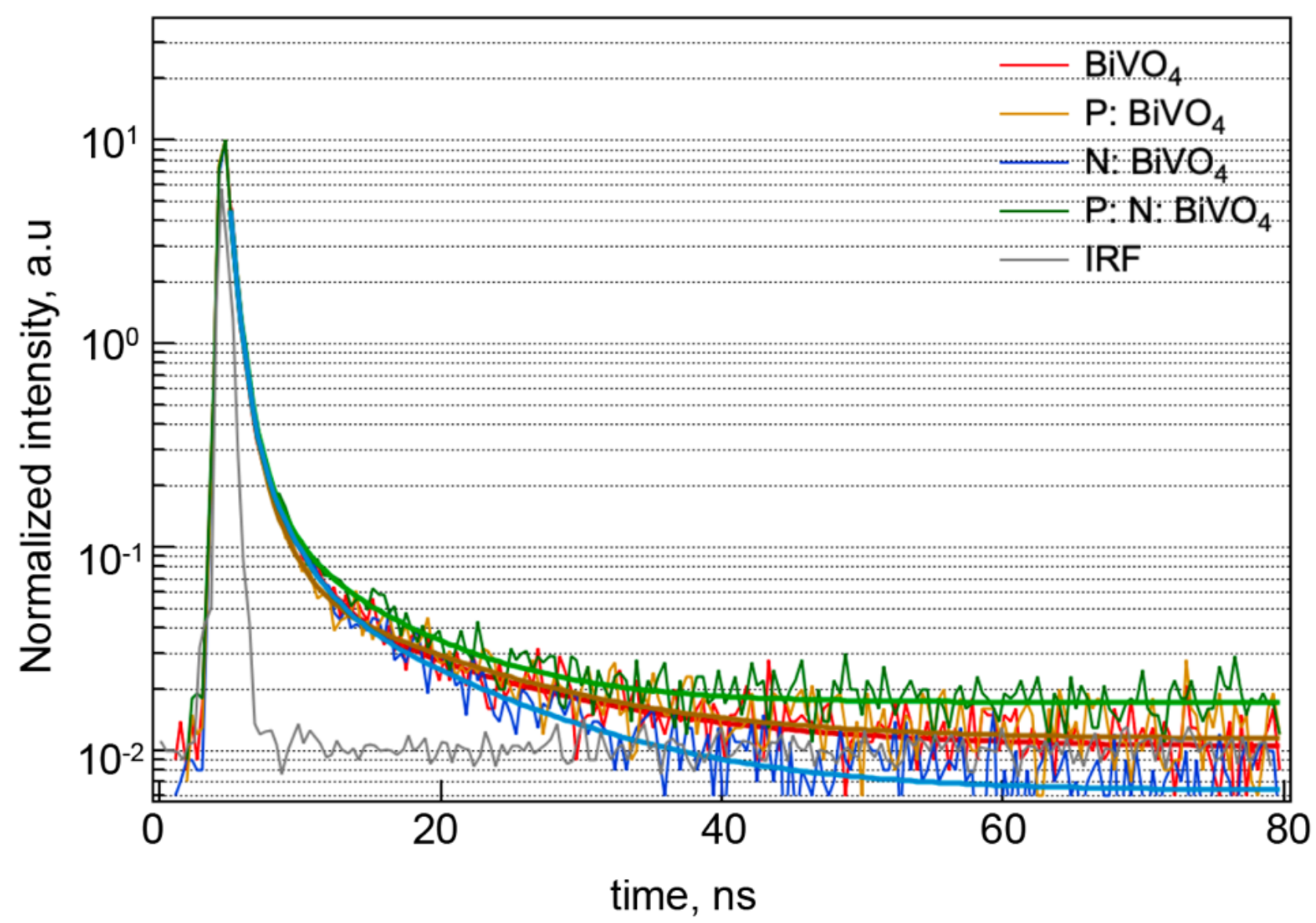

Figure S10. Normalized time-correlated single-photon counting (TCSPC) of $BiVO_4$ particles. The intensity-time traces were background-subtracted, corrected for Rayleigh scattered light, and fitted using eq. 4 with three decay constants.

The following parameters were applied for TCSPC measurements: excitation wavelength: 485 nm, detection wavelength: 490 nm-850 nm, bandwidth: 2 nm, channel: 256, time/channel: 0.3906 nm, excitation repetition rate: 5MHz, time range: 100 ns, instrument response function: ~ 600 ps (PMT, R928P), grating 500 nm Blaze, collection time: 10,000 counts, and background correction was applied using default instrument file. The effect of scattered light (i.e., Rayleigh) was deconvolved using the instrument response function, which was collected using colloidal SiO_2 particles (i.e., Ludox).[3]

The decay behaviors were fitted using three-component decay curves as shown below:

$$I(t) = A_1 e^{\left(-t/\tau_1\right)} + A_2 e^{\left(-t/\tau_2\right)} + A_3 e^{\left(-t/\tau_3\right)} \quad \text{(eq. 4)}$$

Where decay (τ) amplitude (A) values with standard deviation are summarized in Table S2.

Table S2. Fitting parameters of time-correlated single-photon counting (TCSPC) of ensemble $BiVO_4$ particles.

**Sample**	**τ_1(ns)**	**τ_2(ns)**	**τ_3(ns)**	**A_1**	**A_2**	**A_3**	**$\tau_{avg, int}$ (ns)**
**$BiVO_4$**	0.47±0.03	1.90±0.21	12.92±1.77	0.861±0.01	0.125±0.004	0.01±0.002	3.00±0.02
**P:$BiVO_4$**	0.46±0.04	1.65±0.13	10.76±1.01	1.42±0.07	0.317±0.009	0.02±0.002	2.37±0.09
**N:$BiVO_4$**	0.43±0.04	1.34±0.13	9.85±1.01	4.1±0.02	0.391±0.011	0.04±0.002	2.08±0.05
**P:N:$BiVO_4$**	0.36±0.06	1.28±0.29	7.49±0.73	0.943±0.02	0.223±0.005	0.006±0.001	1.22±0.02

The $\tau_{avg, int}$ was calculated using eq. (5)

$$\tau_{avg,int.} = \frac{(A_1\tau_1^2)+(A_2\tau_2^2)+(A_3\tau_3^2)}{(A_1\tau_1)+(A_2\tau_2)+(A_3\tau_3)} \qquad \text{eq. (5)}$$

It is worth noting that the shorter lifetime of the TCSPC results upon doping could be due to the passivation of the deep defects and the formation of shallow radiative centers.

Statistical investigation of the doping effect on PL

To further evaluate the observed differences between PL intensities shown in Figure 5, regression analysis was conducted on individual particle intensity vs. volume. The results revealed $R^2 > 70\%$ (i.e., $BiVO_4$; 72%, P:$BiVO_4$; 76%, N:$BiVO_4$; 81%, and P:N:$BiVO_4$; 83%).

In addition, full population results were obtained by using a multiple comparisons test (i.e., moderate test) where the p-value was calculated using the slope coefficient ($R_{coeff.}$) and slope standard error (RE) of each pair data set (e.g., mean whole particle intensity of $BiVO_4$ vs. V_{BiVO4} in comparison with mean whole particle intensity of P:$BiVO_4$ vs. $V_{P:BiVO4}$, $BiVO_4$ vs. V_{BiVO4} in comparison with mean whole particle intensity of N:$BiVO_4$ vs. $V_{N:BiVO4}$, etc.) For each pair, the following formula was employed to calculate the t-values of the population;

$$T_{obs._{1,2}} = \left| \frac{(R_{Coef.})_1-(R_{Coef.})_2}{\sqrt{(SE_1)^2+(SE_2)^2}} \right| \qquad \text{eq. (6)}$$

where represents mean whole particle intensity of (1) $BiVO_4$ vs. V_{BiVO4}, (2) P:$BiVO_4$ vs. $V_{P:BiVO4}$, (3) $BiVO_4$ vs. $V_{N:BiVO4}$, and (4) P:N:$BiVO_4$ vs. $V_{P:N:BiVO4}$. In each case, n=144.

For example, $T_{obs.1,2}$ is 1.971; and the degree of freedom is (n_1+n_2 –2), 286; then an integration was conducted:

$$T'_{obs_{1,2}} = \int_{1.971}^{\infty} T_{obs.} = 0.975, \text{ then } t = 1 - T'_{obs.1,2} = 1 - 0.97539 = 0.0246$$

Next, calculate the $(p\text{-value})_{1,2}$ using the following formula;

$(p\text{-value})_{1,2} = 2 \times t = 2 \times 0.0246 = 0.049\ (T > |T_{obs.}|)$

p should be less than 0.05 to support a significant difference in the entire population. Our results revealed p-values $(p\text{-value})_{1,2}$: 0.049, $(p\text{-value})_{1,3}$: 0.043, $(p\text{-value})_{1,4}$: 0.015, $(p\text{-value})_{2,3}$: 0.041, $(p\text{-value})_{2,4}$: 0.022, $(p\text{-value})_{3,4}$: 0.014. The results indicate a significant difference in the population.

Correlation between $BiVO_4$ particle squareness and photoluminescence intensity

We also studied whether there is a correlation between the observed PL intensity and the crystal's morphological properties, e.g., dimensions (i.e., volume) or squareness (SQ) of particles, with composition. Figure S11 shows the correlation of the mean PL intensity of particles vs. SQ. The SQ is calculated using equation (7):

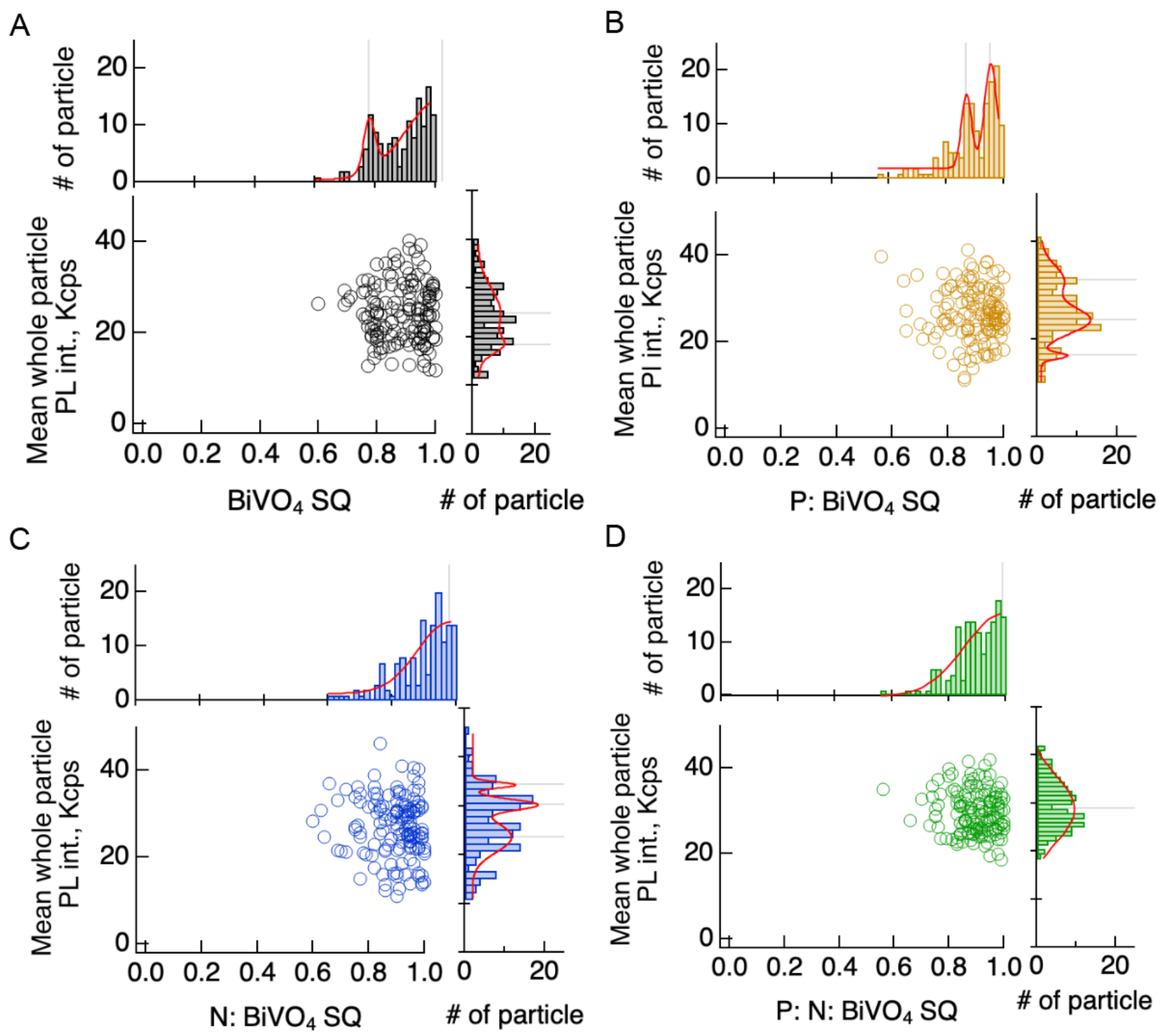

Figure S11. Correlation between $BiVO_4$ particle squareness and photoluminescence intensity. Scatter plot and histogram of the mean whole-particle PL intensity vs. squareness of single particles of (A) $BiVO_4$, (B) $P:BiVO_4$, (C) $N:BiVO_4$, and (D) $P:N:BiVO_4$.

$$SQ = \frac{length\ of\ \ shortest\ whole\ particle\ side}{length\ of\ longest\ whole\ particle\ side} \qquad \text{eq. (7)}$$

When the SQ approaches 1, the particle is highly morphologically symmetric, as the lengths of the sides of the whole particle are identical. One may notice that there are different SQ domains and mean PL intensity for $BiVO_4$ (Figure 11A) and P:$BiVO_4$ (Figure 11B), which were both synthesized via the hydrothermal method. Interestingly, when $BiVO_4$ was treated in the presence of nitrogen, only one SQ domain was observed (Figure S11C); however, multiple populations of the mean whole PL were present.

The particle populations for the mean particle intensity and thickness are represented by the distributions. The $BiVO_4$ and P:$BiVO_4$ SQ show two domains with average sizes of 0.78±0.008 and 0.95±0.004 µm, and 0.87±0.003 and 0.95±0.005 µm; however, N:$BiVO_4$ and P:N:$BiVO_4$ display a single dominant peak at 0.95 ± 0.008 and 0.94 ± 0.005 µm.

Interestingly, P:N:$BiVO_4$ crystals (Figure S11D) show a single population of SQ and mean whole-particle intensity. Indeed, this confirms our observation on the effect of co-doping (Figure 5, main text) on particle PL intensity dependence. This suggests that co-doping promotes more uniform particle morphology and may provide a practical strategy for reducing heterogeneity in semiconductor photocatalysts.

Reference:

(1) Fairley, N.; Fernandez, V.; Richard-Plouet, M.; Guillot-Deudon, C.; Walton, J.; Smith, E.; Flahaut, D.; Greiner, M.; Biesinger, M.; Tougaard, S.; et al. Systematic and collaborative approach to problem solving using X-ray photoelectron spectroscopy. *Appl. Surf. Sci. Adv.* **2021**, *5*, 100112.

(2) Mao, X.; Liu, C.; Hesari, M.; Zou, N.; Chen, P. Super-resolution imaging of non-fluorescent reactions via competition. *Nat. Chem.* **2019**, *11* (8), 687-694.

(3) Margineanu, A. Calibration approaches for fluorescence lifetime applications using time-domain measurements. *Methods in Microscopy* **2025**, *2* (1), 61-71.
